# Genome-Wide Identification and Functional Analysis of Polyamine Oxidase Genes in Maize Reveal Essential Roles in Abiotic Stress Tolerance

**DOI:** 10.3389/fpls.2022.950064

**Published:** 2022-08-04

**Authors:** Yan Xi, Wenjing Hu, Yue Zhou, Xiang Liu, Yexiong Qian

**Affiliations:** Anhui Provincial Key Laboratory of Conservation and Exploitation of Important Biological Resources, College of Life Sciences, Anhui Normal University, Wuhu, China

**Keywords:** polyamine oxidases, polyamines, *Zea mays* L., stress response, functional analysis

## Abstract

Polyamines (PAs) play a critical role in growth and developmental processes and stress responses in plants. Polyamine oxidase (PAO) is a flavin adenine dinucleotide (FAD)-dependent enzyme that plays a major role in PA catabolism. Here, for the first time, *PAO* genes in maize were screened for the whole genome-wide and nine *ZmPAO* genes were identified in this study, named as *ZmPAO1*-*9*. Based on structural characteristics and a comparison of phylogenetic relationships of *PAO* gene families from seven representative species, all nine PAO proteins in maize were categorized into three distinct subfamilies. Further, chromosome location and schematic structure revealed an unevenly distribution on chromosomes and evolutionarily conserved structure features of *ZmPAO* genes in maize, respectively. Furthermore, transcriptome analysis demonstrated that *ZmPAO* genes showed differential expression patterns at diverse developmental stages of maize, suggesting that these genes may play functional developmental roles in multiple tissues. Further, through qRT-PCR validation, these genes were confirmed to be responsive to heat, drought and salinity stress treatments in three various tissues, indicating their potential roles in abiotic stress responses. Eventually, to verify the biological function of *ZmPAO* genes, the transgenic *Arabidopsis* plants overexpressing *ZmPAO6* gene were constructed as a typical representative to explore functional roles in plants. The results demonstrated that overexpression of *ZmPAO6* can confer enhanced heat tolerance through mediating polyamine catabolism in transgenic *Arabidopsis*, which might result in reduced H_2_O_2_ and MDA accumulation and alleviated chlorophyll degradation under heat stress treatment, indicating that *ZmPAO6* may play a crucial role in enhancing heat tolerance of transgenic *Arabidopsis* through the involvement in various physiological processes. Further, the expression analysis of related genes of antioxidant enzymes including glutathione peroxidase (GPX) and ascorbate peroxidase (APX) demonstrated that *ZmPAO6* can enhance heat resistance in transgenic *Arabidopsis* through modulating heat-induced H_2_O_2_ accumulation in polyamine catabolism. Taken together, our results are the first to report the *ZmPAO6* gene response to heat stress in plants and will serve to present an important theoretical basis for further unraveling the function and regulatory mechanism of *ZmPAO* genes in growth, development and adaptation to abiotic stresses in maize.

## Introduction

Polyamines (PAs), largely including spermidine (Spd), spermine (Spm), and putrescine (Put), are small molecular aliphatic amine (Knott et al., 2007; Hussain et al., 2011). PAs have been detected and studied in many actively growing tissues and play crucial roles in nitrogen metabolism (Moschou et al., 2012), dark-induced senescence (Sobieszczuk-Nowicka et al., 2015), flowering (Ahmed et al., 2017), fruit ripening (Handa and Mattoo, 2010; Fortes and Agudelo-Romero, 2018), chloroplast chemical penetration (Ioannidis et al., 2016), callus growth (Liu and Moriguchi, 2007), regulation of protein synthesis (Igarashi and Kashiwagi, 2000), signal molecules (Pál et al., 2019) and especially in the oxidative balance of cells in plants (Murray Stewart et al., 2018). Moreover, increasing studies have elucidated that PAs are involved extensively in various abiotic and biotic stress responses (Alcázar et al., 2006; Kusano et al., 2008; Moschou et al., 2008b; Toumi et al., 2010; Tiburcio et al., 2014; Liu et al., 2015; Corpas et al., 2019). For instance, under salt stress, the application of spermine reduced the harmful effects on the growth and gas exchange of *Tropaeolum majus*, increased the activities of CAT and POD under severe salt stress, and the activities of POD and APX under moderate salt stress (da Silva et al., 2022). Foliar application of putrescine on cucumber alleviated the decrease of stomatal aperture and photosynthesis caused by salt stress treatment, and promoted the growth of cucumber (Ma et al., 2022). Moreover, exogenous putrescine significantly improved ROS scavenging ability and promoted the metabolism of endogenous PAs so as to provide better drought resistance for Cabernet Sauvignon (Zhao et al., 2021). Treatment of seeds of mung bean with PAs can enhance the drought resistance of mung bean plants by accumulating osmoprotectants, improving water status and chlorophyll value, and reducing oxidative damage (Sadeghipour, 2019). Therefore, PAs play a crucial role in maintaining normal growth and development and resisting adverse environmental stresses in plants.

The levels of cellular endogenous PAs largely depend on the dynamic balance of biosynthesis and catabolism of PAs. Polyamine biosynthesis has been thoroughly studied in animals and plants. Generally, the biosynthesis of PAs begins with the synthesis of putrescine. L-ornithine is used for precursor to synthesize Put in animals, whereas L-ornithine and L-arginine serve as precursors to generate Put in plants (Wu et al., 2005; Pegg and Casero, 2011). In plants, arginine decarboxylase (ADC) and ornithine decarboxylase (ODC) are required for catalyzing the synthesis of Put, respectively. Then, Put can be converted into Spd *via* Spd synthase (SPDS). Finally, Spd can be further converted into Spm under the catalysis of Spm synthase (SPMS; Moschou et al., 2008a). In contrast to the anabolism of PAs, the catabolism of PAs in plants is mainly dependent on the catalyzation from two major categories of amine oxidases: diamine oxidases (DAOs) containing divalent Cu^2+^, also known as copper amine oxidases (CuAOs), and flavin-containing polyamine oxidases (PAOs; Pegg and Casero, 2011; Planas-Portell et al., 2013). As far as subcellular localization is concerned, some CuAO and PAO proteins are localized in peroxisomes (Wang et al., 2019). These proteins contain a C-terminal peroxisomal targeting type I (PTS1), a tripeptide consisting of the consensus sequence (S/A/C)(K/R/H)(L/M; Hao et al., 2018). For example, two CuAO (AtCuAO2 and AtCuA03) and three PAO (AtPAO2-AtPAO4) from *Arabidopsis* (Fincato et al., 2011; Planas-Portell et al., 2013), two PAO (CsPAO2 and CsPAO3) from sweet orange (Wang and Liu, 2015), four PAO (SLPAO2-SLPAO 5) from tomato (Hao et al., 2018), and three PAO (OsPAO3-OsPAO5) from rice (Ono et al., 2012) all carry a putative type-I peroxisomal targeting signal in their C-termini. The function of CuAOs is to catalyze the oxidation of Put and cadaverine (Cad) to 4-aminobutyraldehyde, H_2_O_2_ and ammonia (Moschou et al., 2012). In contrast, according to functional differentiation in PAOs involved in polyamine catabolism, PAOs are mainly categorized into two groups in plants. The PAOs of the first group in plants are required for participating in PA terminal catabolism and perform the oxidation and decomposition of Spd and Spm to produce H_2_O_2_, 1, 3-diaminopropane (DAP), and 4-aminobutanal (Spd catabolism) or N-(3-aminopropyl)-4-aminobutanal (Spm catabolism; Angelini et al., 2010; Moschou et al., 2012; Mo et al., 2015), whereas the PAOs in the second group resembles the mammalian Spm oxidases (SMOs), which are mainly involved in back-conversion reactions of PAs by catalyzing Spm to Spd and Spd to Put, in a reverse reaction of PA synthesis and with concomitant production of 3-aminopropanal and H_2_O_2_ (Angelini et al., 2010; Moschou et al., 2012).

In plants, genes encoding PAOs have been identified and characterized in several species including *Arabidopsis* (Fincato et al., 2011), rice (Sagor et al., 2017), sweet orange (Wang and Liu, 2015), tomato (Hao et al., 2018), and tea plant (Li et al., 2020). Most of the identified PAO genes in plants are largely involved in PA back-conversion pathway. For example, five *PAO* genes (*AtPAO1*-*AtPAO5*) in *Arabidopsis* have been revealed to encode PAO proteins (Fincato et al., 2011), all of which are involved in catalyzing PA back-conversion reactions (Tavladoraki et al., 2006; Kamada-Nobusada et al., 2008). Also, a total of seven *PAO* genes (*OsPAO1*-*OsPAO7*) have been identified in rice. Among them, there are four *OsPAO* genes (*OsPAO1*, *OsPAO3*-*OsPAO5*) encoding PAO proteins that are involved in the back-conversion reactions of PAs (Ono et al., 2012). Moreover, there are six *PAO genes* (*CsPAO1*-*CsPAO6*) identified to encode their proteins in sweet orange. Among them, the *CsPAO3* gene has been implicated in participating in the back-conversion reactions of PAs (Wang and Liu, 2015, 2016).

The catabolism of PA generally results in the accumulation of H_2_O_2_ (Liu et al., 2015). H_2_O_2_ can be regarded as a crucial signal molecule at low levels, whereas at high levels it is a toxic compound that causes damage to plant cells (Quan et al., 2008). Therefore, the ratio of PA catabolism to biosynthesis has been considered to be a vital factor in inducing tolerance responses or plant cell death under biotic and abiotic stresses (Moschou et al., 2008a). This suggests that H_2_O_2_ produced by PA catabolism may play a crucial role in maintaining ROS homeostasis, which is closely related to the stress response of plants. For example, the overexpressing of *MdSPDS1* in sweet orange enhanced the activity of PAOs to catalyze polyamine catabolism and thereby result in the accumulation of H_2_O_2_, which likely triggered the hypersensitivity or activation of defense-related genes and eventually significantly reduced the sensitivity of transgenic plants to canker disease (Fu et al., 2011). Under salt stress, the contents of the apoplastic Spm and Spd in maize increased significantly and then the oxidation of free apoplast polyamine catalyzed by polyamine oxidase promoted maize leaf elongation (Rodríguez et al., 2009). In addition, Spd could outflow into the apoplast, and then Spd was decomposed by PAOs to produce H_2_O_2_, which led to stress tolerance response or induction of programmed cell death (PCD) under salt stress in tobacco (Moschou et al., 2008a). Similarly, the ABA signaling pathway integrated PAs and PAOs to regulate the H_2_O_2_ accumulation, which can also cause further stress response or PCD in grape (Toumi et al., 2010). Moreover, H_2_O_2_ produced by Spd oxidation that was catalyzed by PAOs as a signal molecule could trigger the opening of hyperpolarized Ca^2+^ channels to result in Ca^2+^ influx, and thereby regulate the growth of pollen tubes in *Arabidopsis* (Wu et al., 2010). Therefore, H_2_O_2_ produced by PAOs catalyzing the catabolism of PAs may play a vital role in growth, development and response to abiotic stresses in plants *via* modulating the dynamic balance of H_2_O_2_.

Maize (*Zea mays L.*), as one of the most important crop species in the world, has become one of the important model monocot species for functional genomics analysis. It has been of great significance to the study of molecular biology in plants. Meanwhile, maize is a vital part of the food system (Palacios-Rojas et al., 2020). However, biotic and abiotic stresses have significant adverse effects on maize yield and quality. Since PAOs play a vital role in responding to various stress treatments, the study of the roles of PAOs may provide a new way to enhance the stress resistance in plants. However, little is known about identification and functional analysis of polyamine oxidase gene family in maize up to now. In this study, the relevant information on *ZmPAO* genes for the whole genome-wide and the role of *ZmPAO6* under heat stress treatment were further clarified. Taken together, this study may contribute to an in-depth comprehension of the evolution of *PAO* gene family in maize and their crucial roles in abiotic stress resistance.

## Materials and Methods

### Identification of *PAO* Family Genes From Maize

To obtain all putative *PAO* family genes in maize, taking the reported *PAO* gene sequences of *Arabidopsis*, rice, and maize as queries, the programs were used to search the maize genome databases, including the Maize Genome Browser (http://www.maizesequence.org), Phytozome (https://phytozome-next.jgi.doe.gov/), Maize Genetics and Genomics Database (MaizeGDB, http://www.maizegdb.org/), and NCBI (http://www.ncbi.nlm.nih.gov; Zhang et al., 2022). The information about the coding sequence length, amino acid length, and chromosomal localization of each gene was also obtained from NCBI. Furthermore, the molecular weight and theoretical isoelectric point (PI) value of the ZmPAO proteins were reckoned within the online Expasy Bioinformatics Resource Portal (http://web.expasy.org/compute_pi/).

### Sequence Alignment and Phylogenetic Analysis

To determine the phylogenetic relationships of the PAO proteins, full-length amino acid sequences of PAO proteins identified in maize, rice, *Arabidopsis*, barley, tomato, sweet orange, and cotton were aligned by the Clustal W program using their default settings. The information of *PAO* genes in these plants were listed in Supplementary Table S1. Based on the aligned protein sequences, the phylogenetic tree was constructed through the neighbor-joining (NJ) method and examined by bootstrap analyses (1,000 replicates) with MEGA 7.0 (Kumar et al., 2016). Furthermore, the amino acid sequences of PAO proteins in maize were aligned using DNAMAN 7.0 software for multiple protein sequences alignment.

### Analysis of Gene Structure and Conserved Motif of Maize PAO Proteins

The conserved motifs in the full-length amino acid sequences of ZmPAO proteins were identified by the motif analysis tool MEME Suite version 4.0.0 (MEME; http://alternate.meme-suite.org/tools/meme; Bailey et al., 2009). The parameters were set as the following: (1) the maximum number of patterns was set to identify ten motifs; (2) the optimal width of the motif was set between 10 and 50; (3) the rest were executed according to the default parameters (Hu et al., 2021). Furthermore, to analyze the exon and intron structures of *PAO* genes, the structures of *PAO* genes were analyzed by the Gene Structure Display Server database (GSDS, http://gsds.cbi.pku.edu.cn/; Hu et al., 2015).

### Chromosomal Localization and Prediction of cis-Acting Elements in Maize *PAO* Genes

The chromosomal location image of *ZmPAO* genes was accomplished by MapInspect (https://mapinspect.software.informer.com/) based on the information from the Maize Genetics and Genomics Database (http://www.maizegdb.org/). To analyze the cis-regulatory elements of the *ZmPAO* genes in response to abiotic stresses, the promoter sequence in the upstream 2,000 bp of each *ZmPAO* gene start codon was submitted to the PlantCare database (http://bioinformatics.psb.ugent.be/webtools/plantcare/html) to predict stress response and hormone-related cis-acting elements (Chang et al., 2008).

### Expression Profile Analysis of Maize *PAO* Genes in Different Tissues

To explore the spatiotemporal expression patterns of *ZmPAO* genes, the tissue-specific expression patterns of *ZmPAO* genes were analyzed using the genome-wide gene expression atlas of maize inbred line B73 that was reported previously (Sekhon et al., 2011). Microarray analysis was completed to determine the expression patterns of ten representative tissues (seed, root, seedling, stem, shoot tip, silks, leaf, tassel, husk, and endosperm). The signal values for maize *PAO* genes in the ten tissues were list in Supplementary Table S2. The Normalized expression values of the ten tissues were averaged. The gene expression levels were presented as log2 values which were used to generate the heat map of the expressions of *ZmPAO* genes using HEML1.0.3.3 (HeatMap Illustrator, http://ccd.biocuckoo.org/).

### Maize Growth Conditions and Abiotic Stress Treatment

The maize B73 inbred seedlings were grown at 28°C with long-day conditions of 15 h of light and 9 h of dark and an environmental humidity of 60%. After 3 weeks, the seedlings were subjected to heat (42°C), drought, and salt treatments (200 mM NaCl) for 1, 2, 4, and 8 h, respectively. Notably, to impose the drought stress, the roots of the seedlings were pulled out from the soil at 28°C. Three biological replicates were performed for each sample. All collected samples were rapidly frozen in liquid nitrogen and stored at –80°C until RNA extraction.

### Generation of *ZmPAO6* Transgenic *Arabidopsis* Plants

To create *ZmPAO6* overexpression constructs, the coding sequence of *ZmPAO6* was PCR-amplified, with maize B73 cDNA used the templates to clone with high-fidelity polymerase (Trans Start^®^ Fast Pfu DNA Polymerase). The PCR product was purified and cloned into the PHB vector with the EasyGeno Assembly Cloning kit (TIANGEN, China). The vectors were transformed into *Agrobacterium* strain for transformation into *Arabidopsis* by the floral dip method. Seeds of the T_0_ and T_1_ generations were selected on 1/2 MS plates supplemented with hygromycin (30 μg/ml) and confirmed by PCR with primers (Hyg-F: GGTCGCGGAGGCTATGGATGC; Hyg-R: GCTTCTGC GGGCGATTTGTGT). All genes or clones were confirmed by sequencing.

### Heat Tolerance Assay of Transgenic *Arabidopsis*

To evaluate the tolerance for heat stress, the seeds of WT and transgenic *Arabidopsis* homozygous T_3_ lines were grown at 22°C under long-day conditions of 16 h of light and 8 h of dark and an environmental humidity of 60% for 7 days. The three-week-old seedlings were exposed to heat stress (42°C) in the incubator for 36 h. Phenotypic records and physiological parameters analysis were completed at the designed processing time.

### RNA Extraction and Quantitative Real-Time PCR Analysis

According to the manufacturer’s instruction, total RNA was extracted from the collected samples using Trizol RNA isolation (United States of America). For quantitative real-time PCR, cDNA from three distinct biological samples was used for analysis. The PCR condition was performed as follows: pre-denaturation for 15 min at 95°C, 40 cycles at 95°C for 10 s, 55°C for 30 s, and 72°C for 30 s. The gene expression levels were calculated using the 2^−ΔΔCt^ method (Rao et al., 2013). Each experiment was conducted in the form of at least three technologies and biological replication. All the primers in this study were listed in Supplementary Table S3.

### Physiological Parameter Determination

3, 3′-Diaminobenzidine (DAB) was used to dye the leaves to observe the accumulation of H_2_O_2_. The contents of H_2_O_2_ and MDA were measured using the H_2_O_2_ assay kit (A064-1-1) and MDA assay kit (A003-1-1; Nanjing Jiancheng, Nanjing, China). Moreover, the contents of chlorophyll and polyamines were detected by the spectrophotometer and high-performance liquid chromatography, respectively. The experiment was repeated three times at least.

## Results

### Identification and Analysis of *PAO* Genes in Maize

In this study, a total of nine *ZmPAO* genes (namely *ZmPAO1*-*ZmPAO9*) were identified. The related information of *ZmPAO* genes was shown in Table 1, including the chromosome location, the length of coding sequences and corresponding information about the nine proteins. The length of CDS sequences of these putative genes ranged from 504 bp (*ZmPAO7*) to 1,590 bp (*ZmPAO4*), and the amino acid sequences of these proteins ranged from 167 (*ZmPAO7*) residues to 529 (*ZmPAO4*) residues, respectively. The MWs of ZmPAO proteins varied from 19.0 kDa (ZmPAO7) to 57.9 kDa (ZmPAO1) and pIs from 5.21 (ZmPAO3) to 9.89 (ZmPAO7).

**Table 1:** Basic information on *PAO* gene family in maize.

Gene name	Accession number	Genome location coordinates (5′–3′)	CDS (bp)	Protein			Chr
				Length (a.a)	pI	Mol.Wt (Da)	
*ZmPAO1*	Zm00001d024281	63,380,946–63,385,024	1,503	500	5.71	56.37	10
*ZmPAO2*	Zm00001d043681	209,194,699–209,196,394	1,515	504	5.44	53.94	3
*ZmPAO3*	Zm00001d001883	2,622,145–2,625,689	1,473	490	5.21	53.36	2
*ZmPAO4*	Zm00001d026586	149,850,826–149,854,922	1,590	529	5.49	57.95	10
*ZmPAO5*	Zm00001d028172	24,937,595–24,942,561	1,113	370	5.25	42.56	1
*ZmPAO6*	Zm00001d026334	145,108,335–145,113,560	1,449	482	5.60	53.36	10
*ZmPAO7*	Zm00001d037642	139,488,328–139,491,146	504	167	9.89	19.04	6
*ZmPAO8*	Zm00001d036513	97,485,127–97,490,455	1,419	472	5.76	52.39	6
*ZmPAO9*	Zm00001d002266	9,429,838–9,434,928	1,452	483	5.83	53.58	2

### Phylogenetic Analysis and Sequence Alignment of ZmPAO Proteins

**Figure 1:**
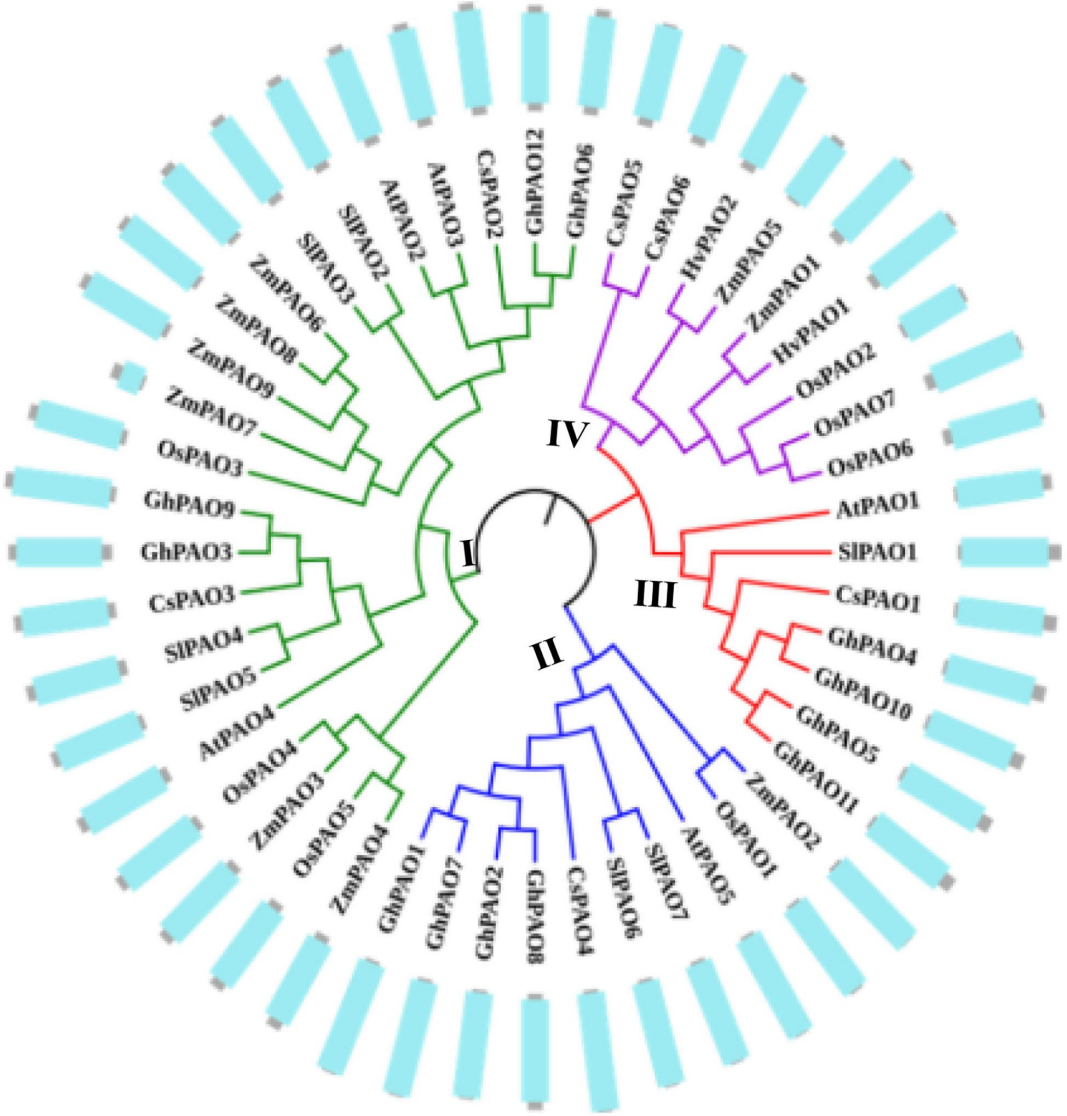
Phylogenetic analysis among the polyamine oxidase (PAO) proteins from seven representative species. The accession numbers of the genes were listed in Supplementary Table 1. The colored branch of the tree indicated that the total PAO proteins were classed into four groups, and nine ZmPAO proteins were classed into three groups according to the amino acid sequence alignment by MEGA 7.0 software with the Neighbor-joining method. The conserved amine-oxidase domains of these proteins were exhibited in the outer ring. Gray represents the length of the amino acid sequence and blue represents the sequence of the conserved amine-oxidase domain.

**Figure 2:**
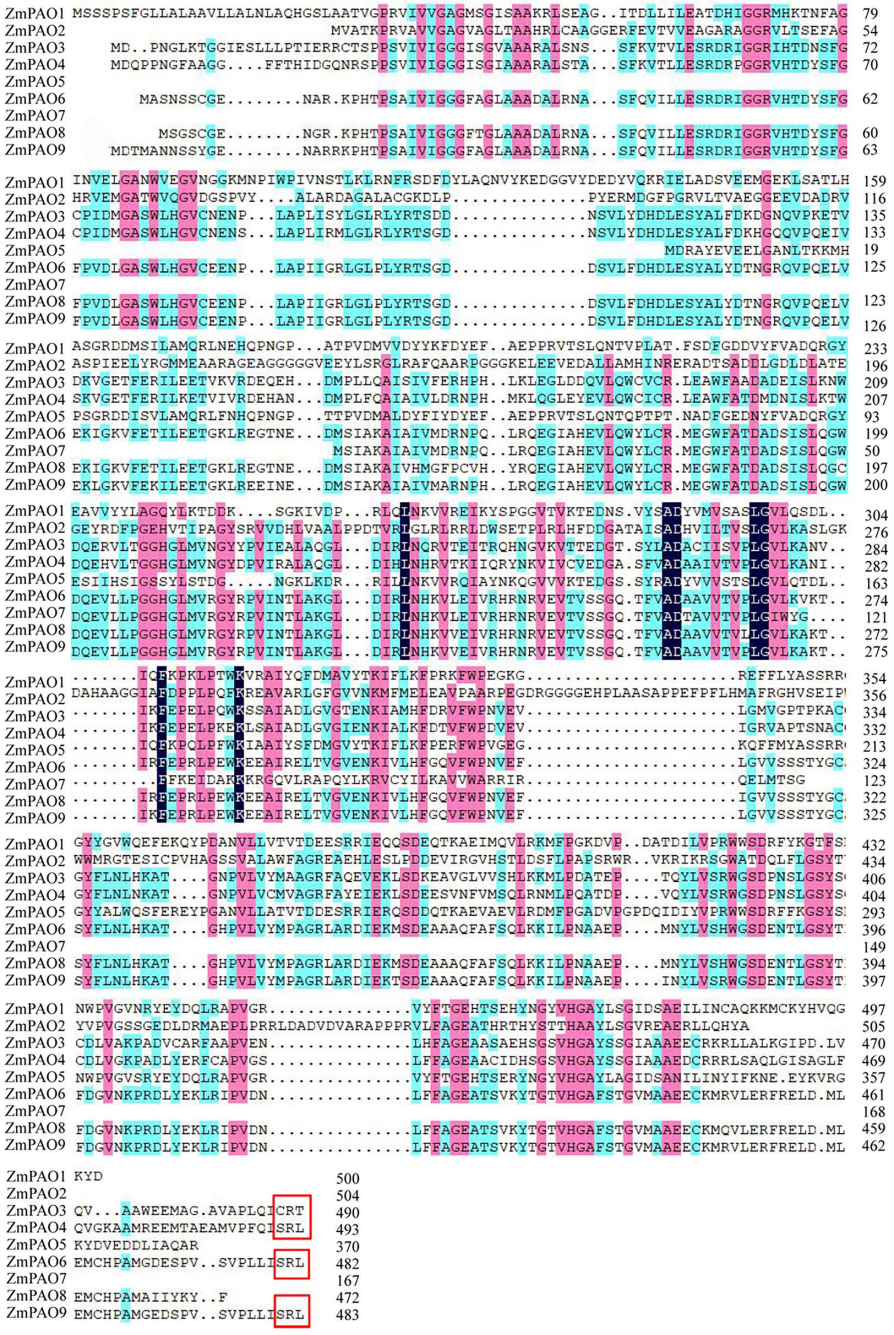
Alignment of the amino acid sequences of maize PAO proteins. Sequence alignment was performed by DNAMAN software. Identical and similar residues are shaded in black or pink and blue background, respectively. The peroxisomal targeting signal of ZmPAO3, ZmPAO4, ZmPAO6, and ZmPAO9 are indicated in the red box.

To identify the candidate PAO proteins in maize, the sequences of PAO proteins from several various species including maize, *Arabidopsis*, rice, barley, tomato, sweet orange and cotton were collected to construct a phylogenetic tree by MEGA 7.0 (Figure 1). The phylogenetic tree revealed that these proteins can be categorized into four groups (I–IV). However, the ZmPAO proteins in maize were only clustered into three groups (I, II, and IV). The Group I was consisted of 22 PAO proteins, including six PAO proteins (ZmPAO3, ZmPAO4, ZmPAO6, ZmPAO7, ZmPAO8, and ZmPAO9). ZmPAO2 protein was clustered into the Group II, and the two remaining proteins including ZmPAO1 and ZmPAO5 were clustered into the Group IV. Multiple sequence alignment was performed using the amino acid sequences of ZmPAO proteins by DNAMAN software (Figure 2). The results demonstrated that ZmPAO4, ZmPAO6, and ZmPAO9 proteins contain SRL sequences. Moreover, ZmPAO3 protein contains CRT sequences, which share high homology with rice OsPAO4 protein. Thus, these results indicated that ZmPAO proteins in maize shared highly conserved homology with other plants in evolution.

### Conserved Motifs of ZmPAO Proteins and Gene Structure of *ZmPAO* Genes

**Figure 3:**
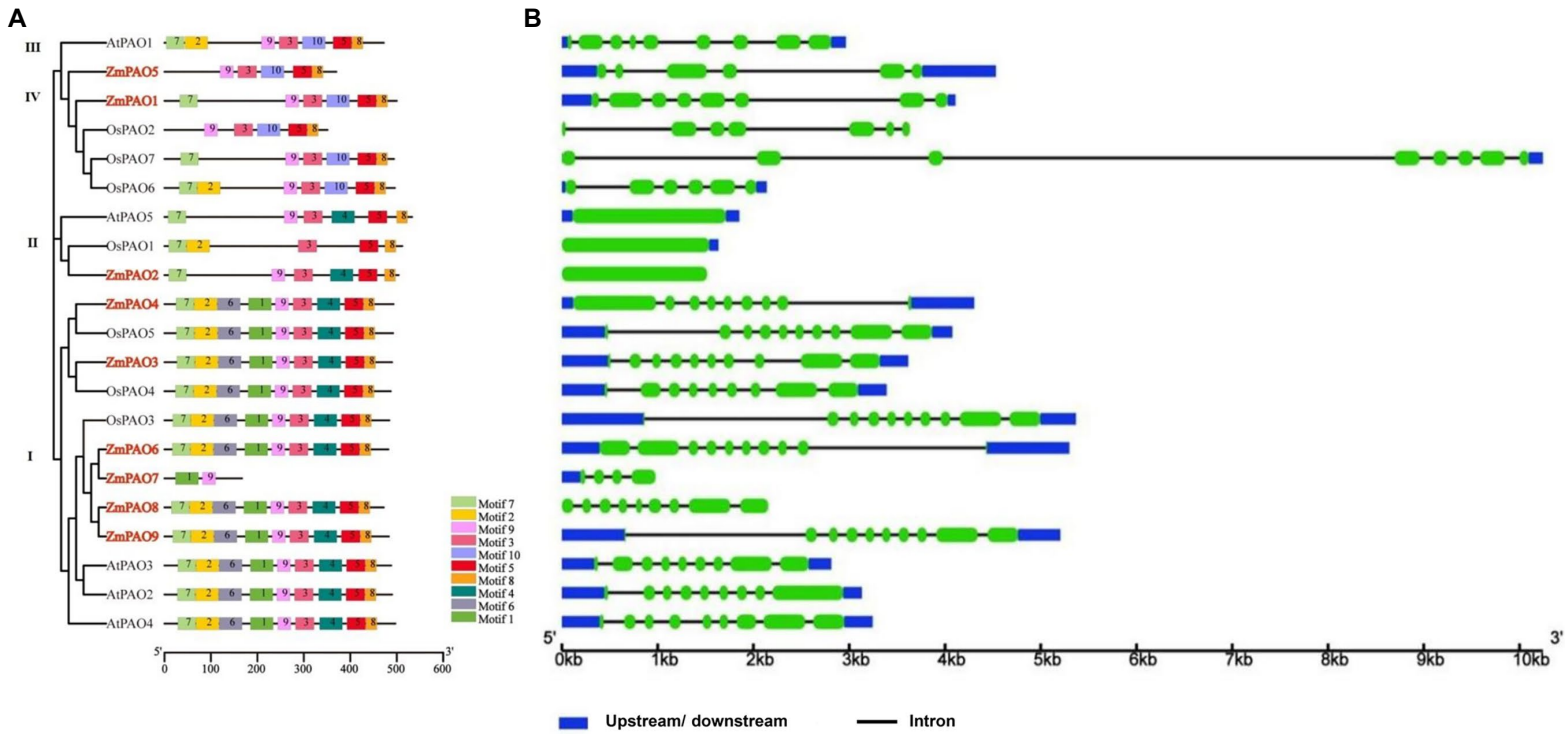
Conserved motifs of PAO proteins and gene structure of *PAO* genes in maize, rice and *Arabidopsis*. **(A)** Distribution of all motifs in maize, rice and *Arabidopsis* were identified by MEME. Different motifs are indicated in various colored boxes. **(B)** Exon/intron structures of *PAO* genes in maize, rice and *Arabidopsis*. Exons are indicated with green boxes, and introns are shown as black lines. The upstream/downstream region of *PAO* genes are indicated in blue boxes. The length of each *PAO* gene inferred by the scale at the bottom.

To further gain more insights into the evolutionarily conserved structure features of *PAO* gene family in maize, a total of ten conserved motifs in ZmPAO, OsPAO, and AtPAO proteins were identified by the MEME website (Figure 3A). In the Group I, all ZmPAO proteins contain the Motifs 1–9 except ZmPAO7 protein, which lacks the Motifs 2–8. All members of the Groups III and IV include the Motifs 3, 5, 8, 9, and 10. Meanwhile, to further perceive the structure of the *PAO* gene family in maize, the intron–exon structures of *ZmPAO* genes were analyzed by GSDS software (Figure 3B). *ZmPAO3*, *ZmPAO6*, and *ZmPAO9* genes all contain nine introns. Secondly, both *ZmPAO4* and *ZmPAO8* genes contain eight introns. Notably, there are some gene structures without introns in *ZmPAO2*, *OsPAO1*, and *AtPAO5* genes. In addition, *ZmPAO1*, *ZmPAO5*, and *ZmPAO7* genes contain 7, 5, and 3 introns, respectively. Overall, these results further demonstrated that the *PAO* gene family in maize exhibited highly conserved structure features during the evolution of plants.

### Chromosomal Location and Gene Duplication of *PAO* Gene Family in Maize

**Figure 4:**
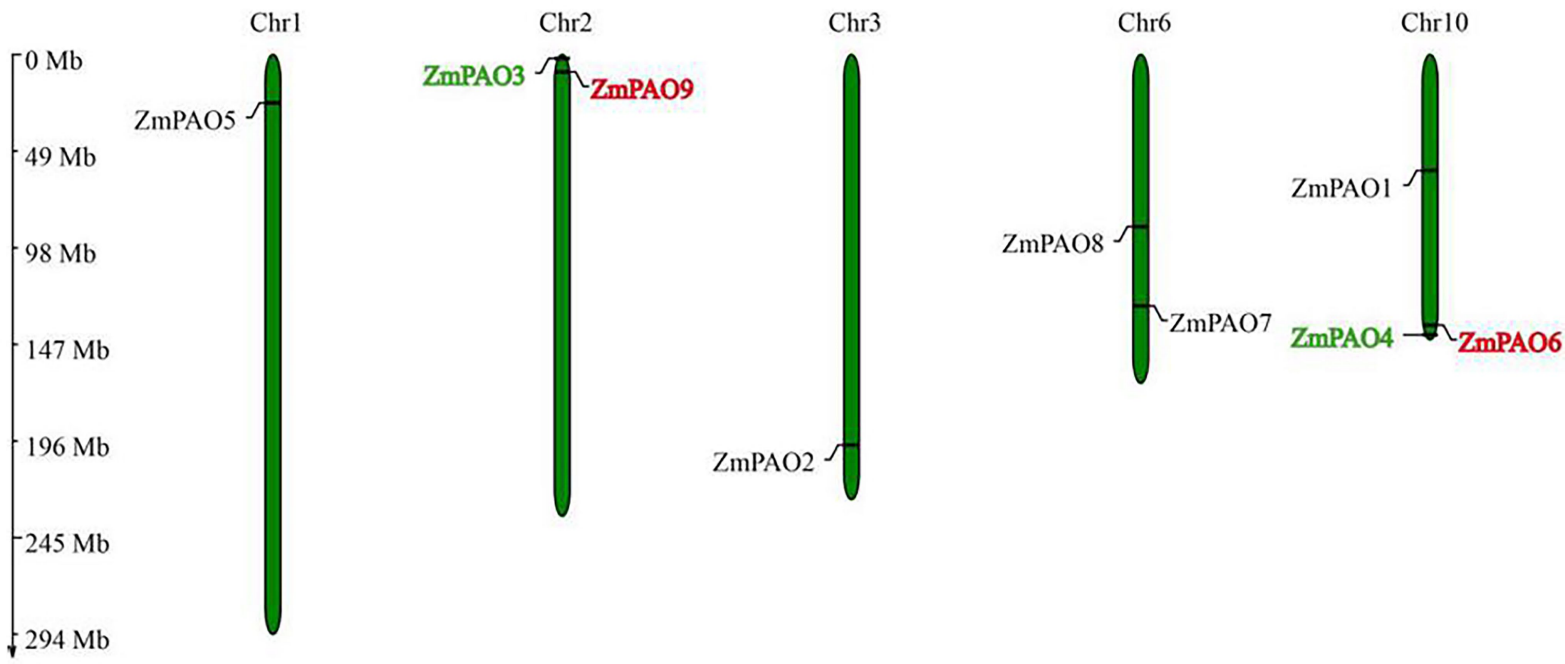
Chromosomal location and gene duplication of the *PAO* gene family in maize. The gene names on each chromosome correspond to the approximate locations of each *ZmPAO* genes. Green and red gene names represent tandem duplicated gene pairs. The size of a chromosome is indicated by its relative length. The chromosome numbers are indicated at the top of each bar. The scale on the left is in megabases.

To generate the chromosome location graphics of *ZmPAO* genes, the physical positions of *ZmPAO* genes were investigated by analyzing the genomic distribution of *ZmPAO* genes on chromosomes in maize. The results indicated that the *ZmPAO* genes were distributed unevenly across five of all the ten chromosomes in the maize genome (Figure 4). Among them, the Chromosome 10 contained the largest number of *ZmPAO* genes with three members (namely *ZmPAO1*, *ZmPAO4*, and *ZmPAO6*), whereas *ZmPAO5* and *ZmPAO1* were located on the Chromosomes 1 and 3, respectively. Moreover, *ZmPAO3* and *ZmPAO9* were distributed on the Chromosome 2. Similarly, *ZmPAO7* and *ZmPAO8* were distributed on the Chromosome 6. Moreover, gene duplication events were investigated to explore the evolutionary patterns of the *PAO* gene family in maize. Two *PAO* gene pairs (*ZmPAO3/ZmPAO4* and *ZmPAO6/ZmPAO9*) were revealed to be involved in maize segmental duplication for they were exhibited to have very high homology in the sequences by analyses of sequence alignment (Figure 2). This result indicated that gene duplication events might have occurred during their process of evolution.

### The cis-Acting Regulatory Elements in the Promoter of Maize *PAO* Genes

**Figure 5:**
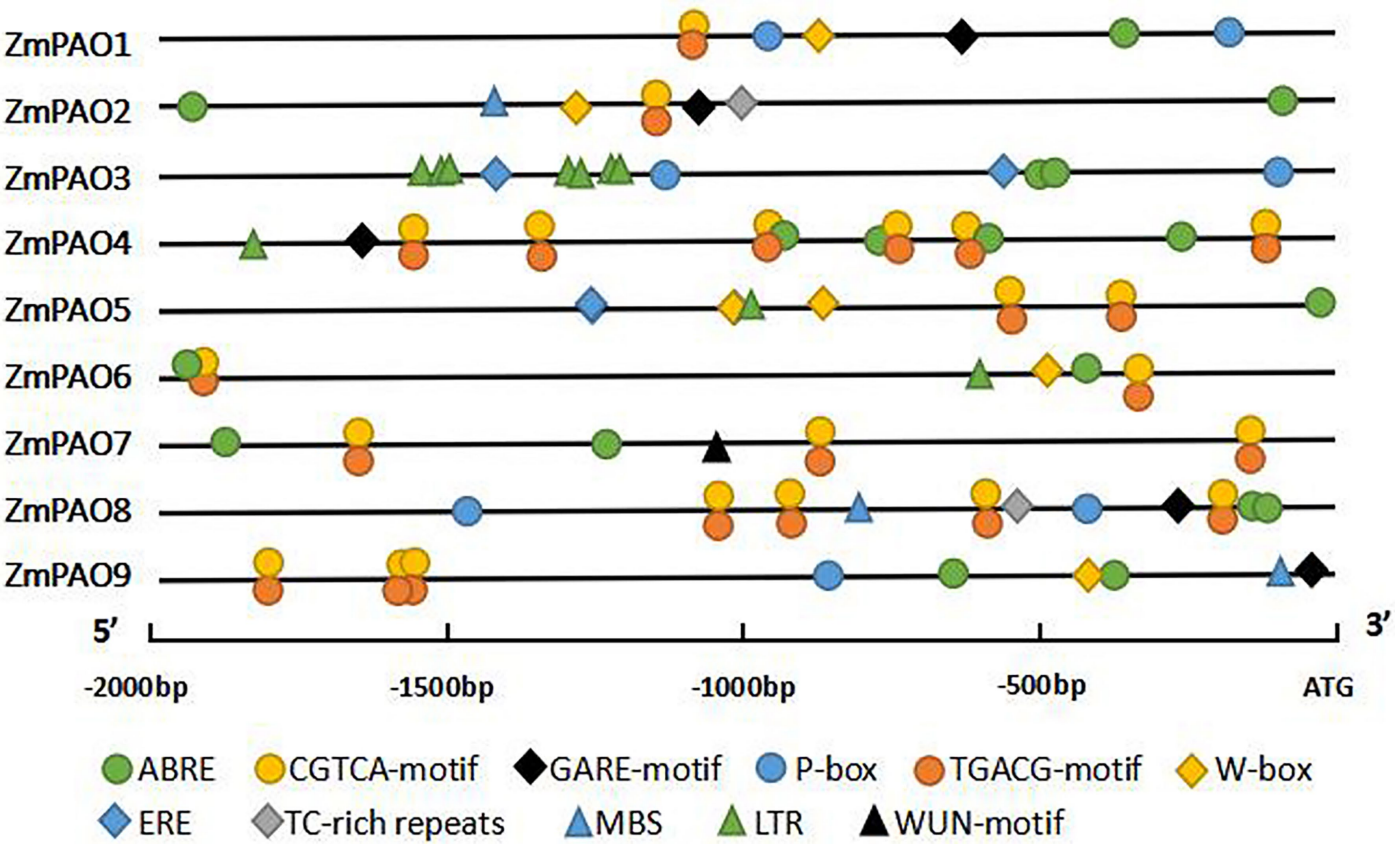
Distribution of major stress-related cis-elements in the promoter sequences of the 9 *ZmPAO* genes. The putative core sequences of these cis-acting elements are represented by different symbols, as shown in the figure key at the bottom. The cis elements distributed on the sense chain and the reverse chain are above and below the black lines, respectively. The 2,000 bp sequences upstream of the initiation codon (ATG) of the PAO genes can be estimated using the scale per 500 bp at the bottom. ABRE, ABA responsive element; ERE, ethylene-responsive element; GARE-motif and P-box, gibberellin responsive element; CGTCA-motif and TGACG-motif, MeJA-responsive element; LTR, low temperature-responsive element; TC-rich repeats, defense and stress-responsive element; WUN-motif, wound-responsive element; MBS, MYB binding site. W-box, wound and pathogen responsive element.

To further explore the potential regulatory mechanisms of *ZmPAO* genes in response to abiotic stresses in maize, the promoter sequences were analyzed using the PLACE database to identify cis-regulatory elements in the promoter region. The results indicated that 11 types of stress- and hormone-related cis-acting regulatory elements were detected in the promoters of maize *PAO* genes: five hormone-related elements (ABRE, CGTCA-motif, TGACG-motif, GARE-motif, and P-box) and six stress-related elements (TC-rich repeats, MBS, LTR, ERE, WUN-motif, and W-box; Figure 5). It is worth noting that the ABRE (a cis-element involved in ABA responsiveness) were detected in the promoter regions of all the *ZmPAO* genes. Except for *ZmPAO3*, MeJA-responsive elements were discovered in the promoters of the other eight *ZmPAO* genes. In addition, ethylene-responsive element (ERE) was uncovered in the promoter regions of the *ZmPAO3* and *ZmPAO5* genes. Low temperature-responsive element (LTR) was predicted in the promoter regions of the *ZmPAO3*, *ZmPAO4*, *ZmPAO5*, and *ZmPAO6* genes, while WUN-motif (wound-responsive element) was discovered in the promoter region of the *ZmPAO7* gene. Meanwhile, the promoter regions of most *ZmPAO* genes contain the W-box element. TC-rich repeats as defense and stress-responsive elements were predicted to exist in the promoters of the *ZmPAO2* and *ZmPAO8* genes. This result indicated that *ZmPAO* genes might be mainly involved in hormone regulation and adversity stress.

### Analysis of Microarray Expression Profile of Maize *PAO* Genes

**Figure 6:**
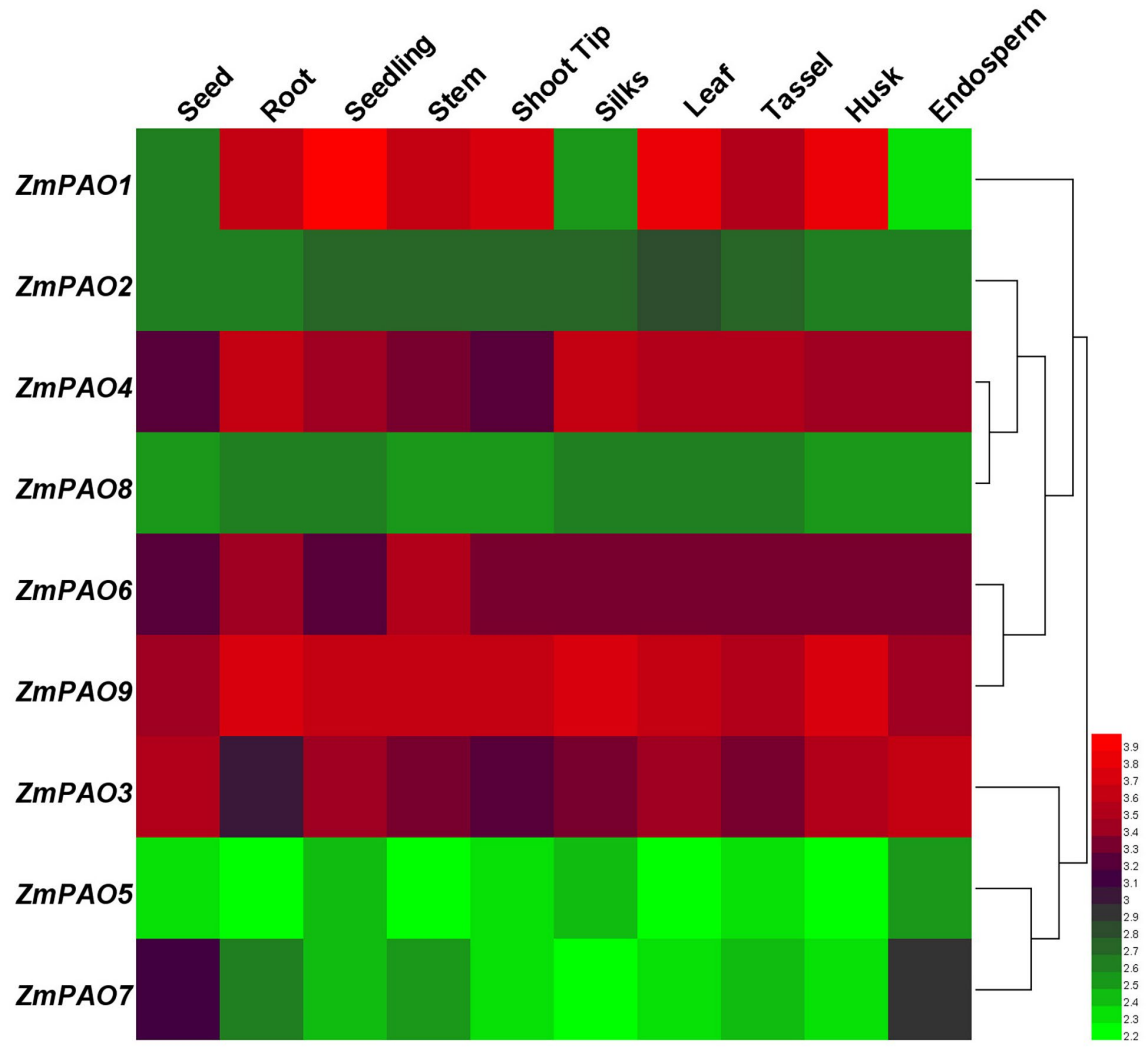
Hierarchical clustering of expression profiles of *ZmPAO* gene family in ten tissues. Ten tissues from different developmental stages including seed, root, seedling, stem, shoot tip, silks, leaf, tassel, husk, and endosperm were investigated. The different tissues are marked at the top of each channel. Cluster dendrograms are displayed on the right. The color scale representing the value of the log 2 signal is shown on the right. Green represents low expression levels and red indicates high expression levels of the transcript abundance.

To further investigate the roles of maize *PAO* genes in plant growth and development, the expression profiles analysis was accomplished using the available transcriptomic data of maize B73 (Sekhon et al., 2011). The signal values for all these maize *PAO* genes are given in Supplementary Table S2. The expression heat map of all the *ZmPAO* genes from ten developmental stages was constructed using Helm software (Figure 6). The results revealed that all nine *ZmPAO* genes were expressed at varying levels in all tissues. Compared with the other *ZmPAO* genes examined, the transcripts of the *ZmPAO9* gene showed relatively high expression levels at all stages, while the transcripts of the *ZmPAO5* gene maintained low expression levels in all tissues. Interestingly, some tissue-specific genes were discovered, such as high expression levels of the *ZmPAO1* gene in seedlings and high expression levels of the *ZmPAO6* gene in roots and stems, suggesting that those genes may play a vital role in plant growth and development processes of specific tissues. In general, the expression patterns in the same paralogous gene pairs (*ZmPAO3*/*ZmPAO9*, *ZmPAO4*/*ZmPAO6*) were similar, indicating they might be formed by segmental duplication and retain their functions. It is noteworthy that the expression patterns of four *ZmPAO* genes (*ZmPAO2*, *ZmPAO5*, *ZmPAO8*, and *ZmPAO9*) in all tissues were relatively stable, implying that these genes might be involved in the basic metabolism. Furthermore, the hierarchically clustered heat map could be divided into two clusters. The first cluster included six members (*ZmPAO1*, *ZmPAO2*, *ZmPAO4*, *ZmPAO6*, *ZmPAO8*, and *ZmPAO9*), while the second cluster included three members (*ZmPAO3*, *ZmPAO5*, and *ZmPAO7*). Notably, the changing trend of gene expression levels of the members of the same cluster was similar in the ten tissues. Overall, the results demonstrated that these identified *ZmPAO* genes showed differential expression patterns at diverse developmental stages of maize, suggesting that these genes may function in multiple tissues.

### Expression Profile Analyses of Maize *PAO* Genes Under Abiotic Stress Treatment

**Figure 7:**
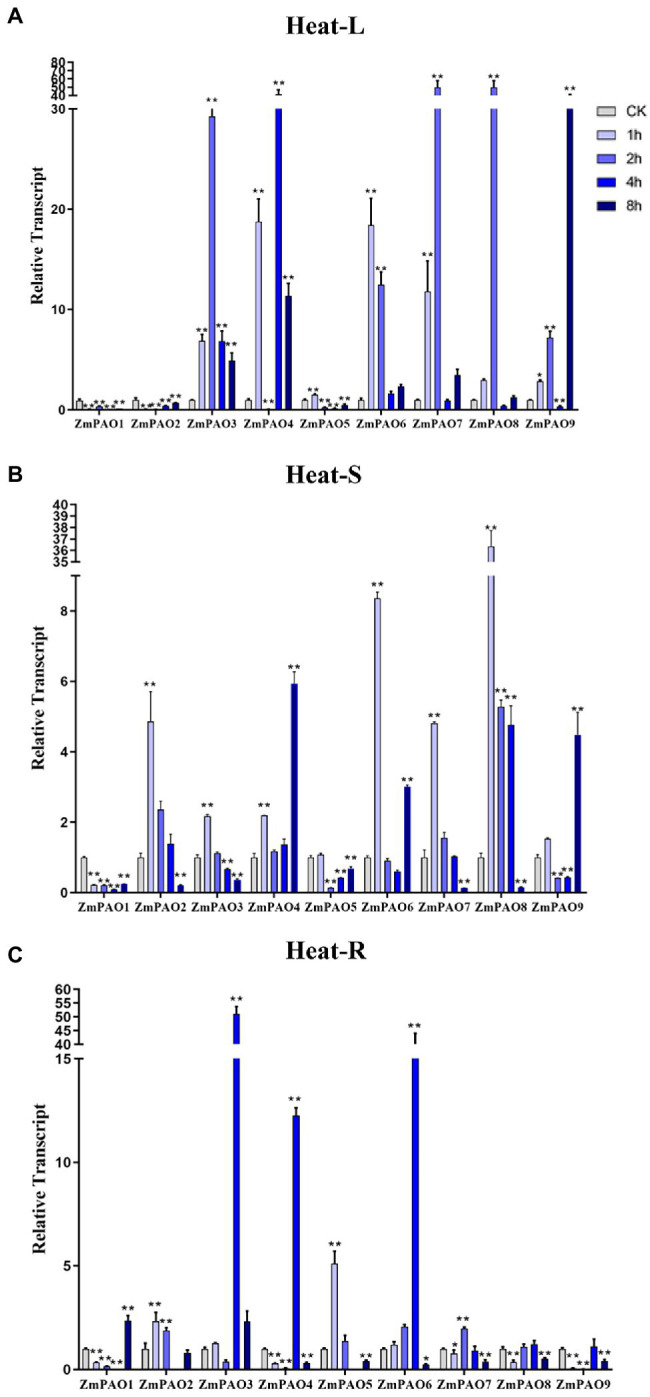
Expression profiles of *ZmPAO* genes under heat stress treatment in different tissues. qRT-PCR data was normalized using *ZmActin* gene. **(A–C)** Expression profiles of *ZmPAO* genes under heat treatment (42°C) in leaves, stems, and roots, respectively. Error bars are caused by three biological replicates. Asterisks above the error bars represent statistical significance using one-way ANOVA and a Fisher’s least significant difference (LSD). ^*^ Significantly different at *p* < 0.05; ^**^ significantly different at *p* < 0.01.

**Figure 8:**
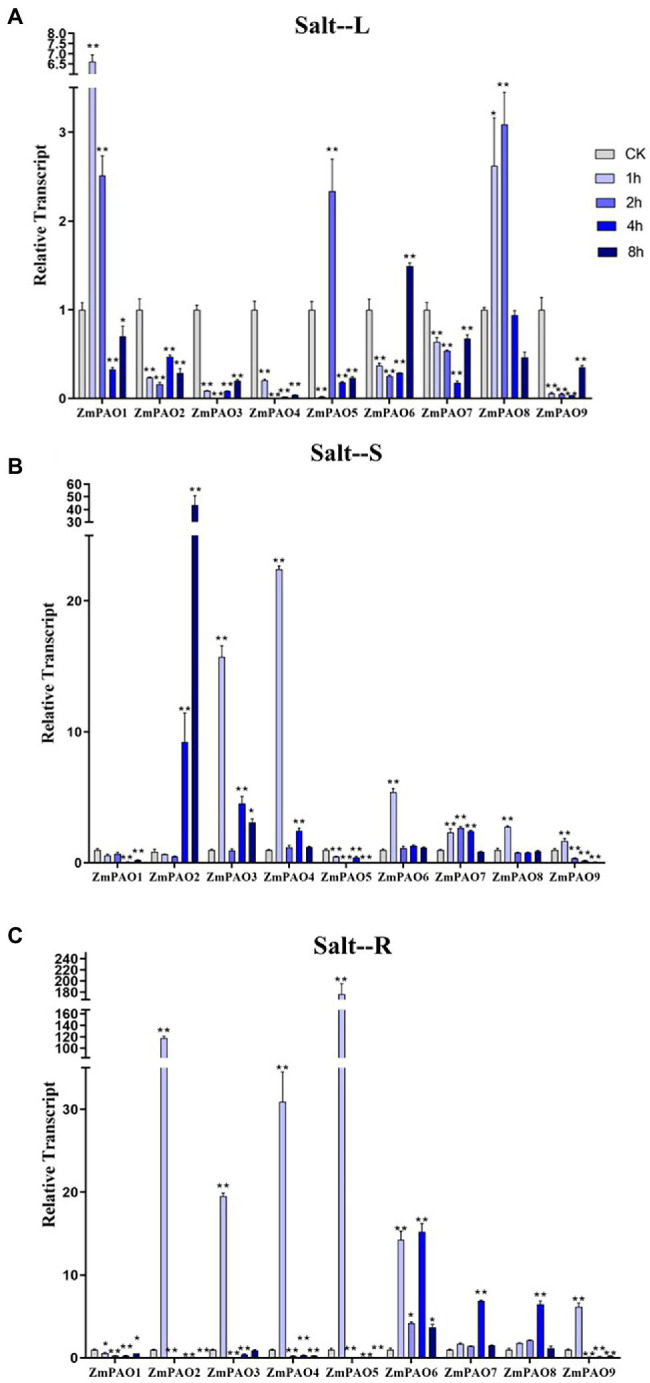
Expression profiles of *ZmPAO* genes under salt stress treatment in different tissues. qRT-PCR data was normalized using *ZmActin* gene. **(A–C)** Expression profiles of *ZmPAO* genes under salt treatment with 200 mM NaCl in leaves, stems, and roots, respectively. Error bars are caused by three biological replicates. Asterisks above the error bars represent statistical significance using one-way ANOVA and a Fisher’s LSD. ^*^ Significantly different at *p* < 0.05; ^**^ significantly different at *p* < 0.01.

**Figure 9:**
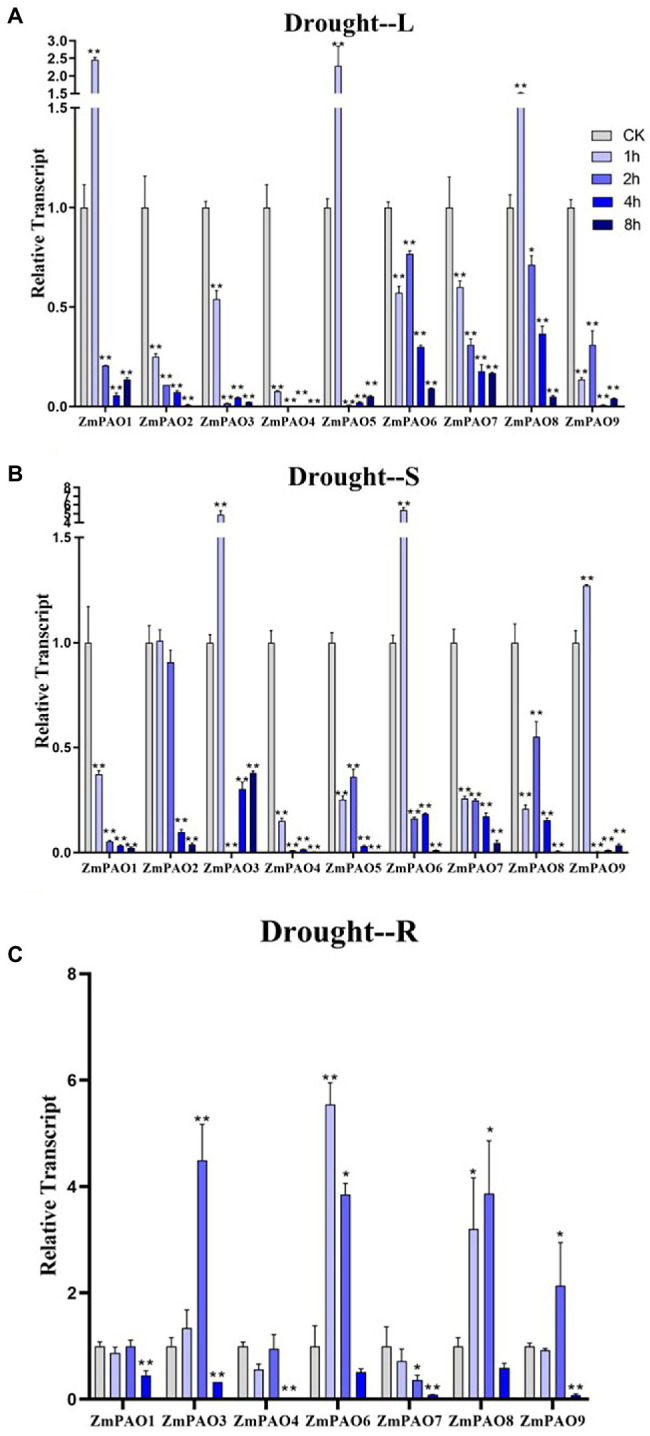
Expression profiles of *ZmPAO* genes under drought stress treatment in different tissues. qRT-PCR data was normalized using *ZmActin* gene. **(A–C)** Expression profiles of *ZmPAO* genes under drought stress in leaves, stems, and roots, respectively. Error bars are caused by three biological replicates. Asterisks above the error bars represent statistical significance using one-way ANOVA and a Fisher’s LSD. ^*^ Significantly different at *p* < 0.05; ^**^ significantly different at *p* < 0.01.

To survey if these predicted genes were expressed in maize and to further confirm their stress-responsiveness to abiotic stresses, the expression profiles of the nine *ZmPAO* genes in roots, stems, and leaves under heat, salt, and drought treatments were determined by qRT-PCR (Figure 7). The results indicated that the expression levels of the nine *ZmPAO* genes were different in three distinct tissues under varied abiotic stress conditions. The expression levels of most *ZmPAO* genes changed significantly before and after abiotic stress treatments, which indicated that these genes were responsive to these stress treatments and could be involved in distinct stress response pathways. Firstly, the detection of gene expression levels at different time points after heat stress revealed that most *ZmPAO* genes were sensitive to heat stress (Figure 7A). The gene expression levels of *ZmPAO3*, *ZmPAO4*, and *ZmPAO6* changed significantly in all tissues after heat stress, which indicated that they could be involved in heat stress. Notably, the expression of *ZmPAO1* was down-regulated at all time points in other tissues except that it was up-regulated at 8 h after heat stress in the roots. Moreover, most members exhibited low expression levels in the stems (Figure 7B). Secondly, under the treatment of 200 mM NaCl concentration, the expression level of *ZmPAO* gene changed significantly at different time points (Figure 8). In the leaves (Figure 8A), the expression levels of *ZmPAO2*, *ZmPAO3*, *ZmPAO4*, *ZmPAO7*, and *ZmPAO9* genes were significantly down-regulated at all time points. In the stems (Figure 8B), the expression levels of *ZmPAO1* and *ZmPAO5* genes under salt treatment were both down-regulated compared with the control group. In the roots (Figure 8C), the expression levels of 6 genes (*ZmPAO2*-*ZmPAO6* and *ZmPAO9*) were significantly up-regulated in the early stage of salt stress. Moreover, the expression level of *ZmPAO1* gene was inhibited during the whole salt stress period, and only the expression level of *ZmPAO6* gene was higher than the control group during the salt stress period. Finally, under drought treatment, the expression changes of *ZmPAO* genes at different times were analyzed (Figure 9). In the leaves (Figure 9A), the expression levels of *ZmPAO2*, *ZmPAO3*, *ZmPAO4*, *ZmPAO6*, *ZmPAO7*, and *ZmPAO9* genes decreased as a whole, among which the expression levels of *ZmPAO3* and *ZmPAO4* genes decreased the most. In the stems (Figure 9B), the expression levels of most *ZmPAO* genes were decreased under drought treatment compared with the control group. In the roots (Figure 9C), the expression levels of *ZmPAO3* and *ZmPAO8* genes were similar, which were slowly up-regulated at the beginning of drought treatment, reached the maximum at 2 h, and decreased at 4 h. Taken together, the results demonstrated that most of these predicted genes of the *PAO* gene family in maize were responsive to various abiotic stress treatments, suggesting their potential roles in abiotic stress responses.

### Genetic Transformation and Overexpression Analysis of *ZmPAO6* Gene in *Arabidopsis*

The *ZmPAO6* clone map and the double enzyme (BamH I and Xba I) digestion map of the recombinant plasmid were shown in Supplementary Figures 1A,B, respectively. To investigate the role of *ZmPAO6* gene under heat stress, the recombinant vector with the *ZmPAO6* gene was transformed into the *Arabidopsis* line using *Agrobacterium*-mediated method by dipping the *Arabidopsis* floral to obtain the overexpressed *Arabidopsis* plants. Consequently, a total of 14 transgenic lines were generated in this study (Supplementary Figure 2). In addition, according to the analysis of the phylogenetic tree, the *ZmPAO6* gene is located in the Group I, and its orthologous genes in *Arabidopsis* (*AtPAO2* and *AtPAO3*) and rice (*OsPAO3*) are both confirmed to be located in peroxisomes and participate in the reverse conversion pathway of polyamine catabolism. Thus, the amino acid sequence of *ZmPAO6* gene and these three orthologous *PAO* genes were compared, and the results elaborated that the sequence homology of both Z*mPAO6* and *OsPAO3*, *AtPAO2*, or *AtPAO3* are 88.28, 69.29, and 68.28%, respectively (Supplementary Figure 3). Peroxisome targeting sequence (PSTI) SRL was also presented at the end of ZmPAO6 protein. The function of *ZmPAO6* gene may resemble the orthologous *OsPAO3* gene, which encompasses high homology, locates in subcellular peroxisome and participates in the reverse conversion pathway of decomposing PAs. Thus, this result indicated that the *ZmPAO6* gene may function in growth, development and adversity stress in maize.

### Detection of Polyamine and Hydrogen Peroxide Contents in Transgenic Plants

**Figure 10:**
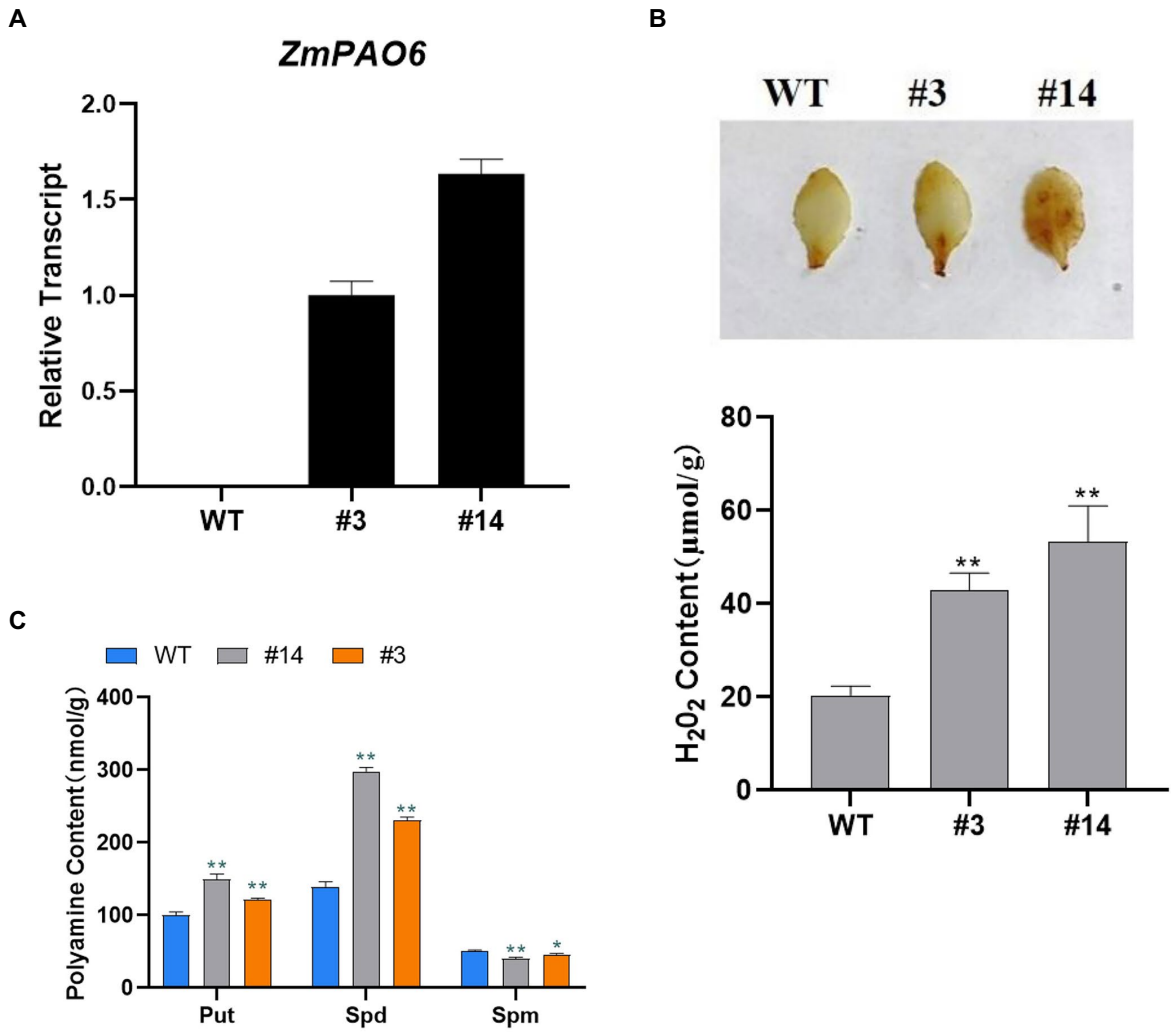
Overexpression of *ZmPAO6* gene leads to changes in characteristics of transgenic *Arabidopsis*. **(A)** Molecular characterization of transgenic lines by qRT-PCR analysis. **(B)** H_2_O_2_ contents in WT and transgenic lines. **(C)** PAs contents in WT *Arabidopsis* and transgenic lines detected by HPLC. Error bars are caused by three biological replicates. Asterisks above the error bars represent statistical significance using one-way ANOVA and a Fisher’s LSD. ^*^ Significantly different at *p* < 0.05; ^**^ significantly different at *p* < 0.01.

To further confirm the function of *ZmPAO6* gene in transgenic *Arabidopsis*, two representative transgenic lines (#3 and #14) were selected and identified for further functional analysis (Figure 10A). According to the metabolic pathways and products of PAs, PAs can be decomposed by polyamine oxidase to produce H_2_O_2_ (Moschou et al., 2012). The leaves of the WT *Arabidopsis* and transgenic *Arabidopsis* lines were firstly stained by DAB, and the H_2_O_2_ content was detected as shown in Figure 10B. The leaves of transgenic lines # 3 and # 14 were stained in a darker color and larger area compared with the WT *Arabidopsis*, indicating that the content of H_2_O_2_was higher in transgenic plants, which may be due to the overexpression of *ZmPAO6* in *Arabidopsis* may promote the catabolism of PAs. Therefore, these results suggested that *ZmPAO6* gene may be involved in the catabolism of plant polyamines. To confirm the effect of overexpressed *ZmPAO6* gene on its polyamine catabolism in transgenic *Arabidopsis* plants, the content changes of PAs in the WT and transgenic lines with the overexpression of *ZmPAO6* gene were determined by high-performance liquid chromatography, respectively (Figure 10C). The contents of Put and Spd in the transgenic lines were higher than those of the WT *Arabidopsis*, especially the content of Spd. Meanwhile, compared with the WT *Arabidopsis*, the content of Spm in the transgenic lines were reduced and the line #14 had the lowest content of Spm. The results further revealed that the overexpressed *ZmPAO6* gene could be involved in decomposing Spm into Spd and further converting Spd into Put through catalyzing the back-conversion reactions in the catabolism of polyamines in the transgenic *Arabidopsis* plants. Therefore, these results suggested that the *ZmPAO6* gene may be implicated in promoting specific PA degradation mainly *via* participating in back-conversion reaction of PAs.

### Overexpression of *ZmPAO6* Gene Enhances Heat Tolerance in Transgenic *Arabidopsis*

**Figure 11:**
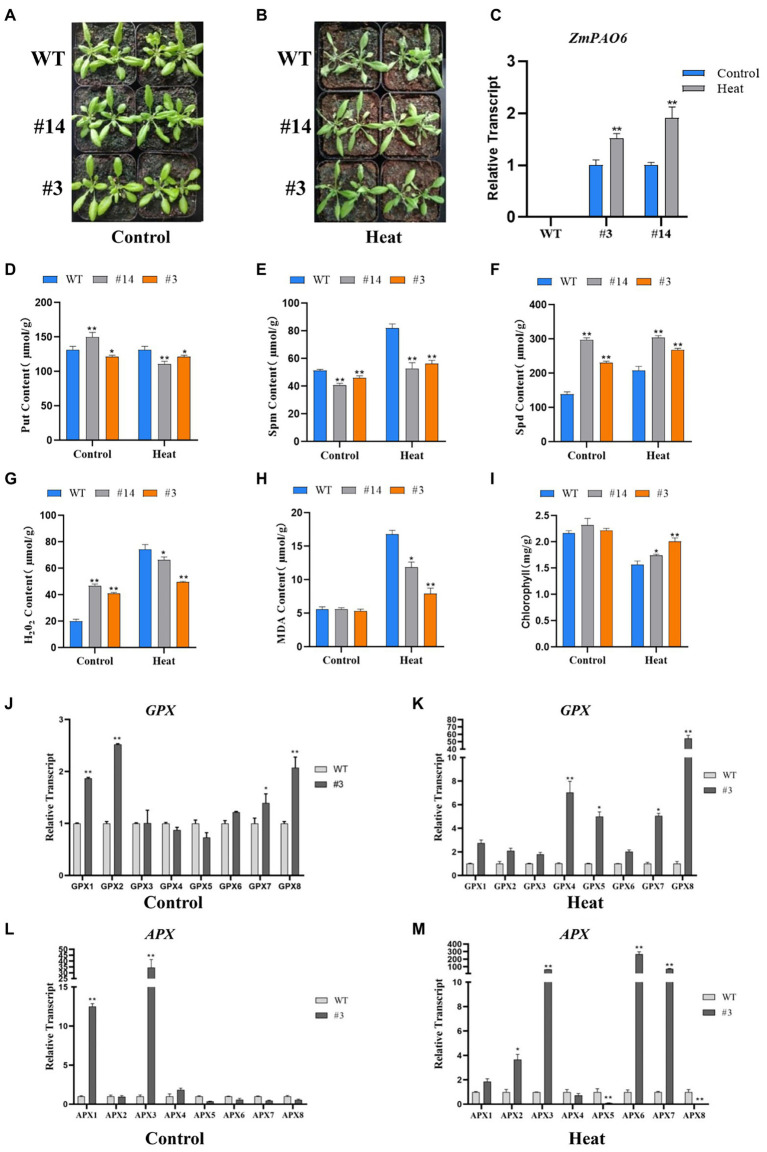
Overexpression of *ZmPAO6* gene enhances heat tolerance in *Arabidopsis*. **(A,B)** The growth status of WT *Arabidopsis* and transgenic lines during heat stress treatment. **(C)** Expression profiles of *ZmPAO6* gene in WT *Arabidopsis* and the transgenic lines under control and heat stress treatment. **(D–F)** Analysis of Put, Spm and Spd contents in WT *Arabidopsis* and the transgenic lines under control and heat stress treatment. **(G–I)** The change of H_2_O_2_, MDA, and Chlorophyll contents in seedlings leaves. **(J–M)** The expression levels of *AtGPX1-8* and *AtAPX1-8* under control and heat stress treatment. Error bars are caused by three biological replicates. Asterisks above the error bars represent statistical significance using one-way ANOVA and a Fisher’s LSD. ^*^ Significantly different at *p* < 0.05; ^**^ significantly different at *p* < 0.01.

To detect the tolerance of transgenic *Arabidopsis* in response to heat stress, 3-week-old seedlings were chosen and treated for 36 h at 42°C. From the phenotypic observation, the leaves of WT *Arabidopsis* showed obvious withering and curling compared with those of the transgenic *Arabidopsis* lines (Figures 11A,B). However, the leaves of the line #14 were slightly curled, and the leaves of the line #3 were not significantly damaged. The results indicated that the damage of transgenic plants under heat stress was lower than that of the WT *Arabidopsis*. The expression level changes of *ZmPAO6* gene in the lines # 3 and # 14 before and after heat stress treatment were determined by qRT-PCR (Figure 11C). The results revealed that the expression levels of *ZmPAO6* gene in the lines # 3 and # 14 were up-regulated significantly after heat stress, indicating that the overexpression of *ZmPAO6* gene could enhance thermotolerance of transgenic *Arabidopsis* plants.

After heat stress treatment, the PA contents of the WT and transgenic plants were further determined (Figures 11D–F). Compared with the control, the Put content of transgenic plants decreased slightly or remained unchanged, while the Put content of WT plants increased, which were slightly higher than those of transgenic plants (Figure 11D). The Spm content of the WT and transgenic *Arabidopsis* increased, but the increasing of Spm content in WT *Arabidopsis* was higher than that in transgenic *Arabidopsis* plants (the lines # 3 and # 14; Figure 11E). In addition, the Spd content of all plants increased after heat treatment. Notably, the Spd content of the WT *Arabidopsis* were much lower than those of the transgenic plants (Figure 11F). This result further indicated that *ZmPAO6* gene is likely to mainly participate in back-conversion reaction of PAs. Furthermore, to explore molecular mechanism of heat tolerance of transgenic *Arabidopsis* in response to heat stress, the analyses of H_2_O_2,_ MDA and chlorophyll contents were further determined according to the physiological methods (Figures 11G–I). The results demonstrated that the contents of H_2_O_2_ in the WT and transgenic plants increased under heat stress treatment compared with those under the control condition. However, the content of H_2_O_2_ in the WT *Arabidopsis* was obviously higher than those in the transgenic *Arabidopsis* lines. Therefore, the *ZmPAO6* gene could be involved in modulating heat-induced H_2_O_2_ accumulation through the catabolism pathways of polyamines in transgenic *Arabidopsis* plants to maintain the dynamic balance of H_2_O_2_ in the cell of plants and thereby enhancing thermotolerance of plants. In addition, the MDA contents both in WT *Arabidopsis* and in transgenic plants also increased after heat stress treatment, but the MDA content of WT *Arabidopsis* was higher than those of the transgenic *Arabidopsis* lines, indicating that the cells in the WT *Arabidopsis* were damaged more deeply than those in the transgenic *Arabidopsis* plants. Notably, the chlorophyll contents of the WT and transgenic *Arabidopsis* lines decreased after heat stress treatment. However, the chlorophyll contents of the transgenic *Arabidopsis* lines were higher than those of the WT *Arabidopsis*, suggesting that an enhanced heat resistance could occur in the transgenic *Arabidopsis* lines compared with in the WT *Arabidopsis*.

To further confirm the role of the *ZmPAO6* gene in regulating the dynamic balance of H_2_O_2_ in the transgenic *Arabidopsis* plants, the expression levels of some antioxidase genes (*AtGPX1-8* and *AtAPX1-8*) published in the previous studies after heat stress treatment were further determined in this study (Figures 11J–M). Under the control condition, the expression levels of most of *AtGPX* genes in the transgenic *Arabidopsis* plant (line #3) were higher than that of the WT *Arabidopsis*. However, under heat stress treatment, the expression levels of all *AtGPX* genes in the transgenic *Arabidopsis* plant (line #3) were significantly enhanced. Notably, the expression level of *AtGPX8* gene in the transgenic *Arabidopsis* plant was higher at more than 60 times than that in the WT Arabidopsis. Likewise, in the control group, the expression levels of only three genes (*AtAPX1*, *AtAPX3*, and *AtAPX4*) in the transgenic *Arabidopsis* plant were significantly higher than those in the WT *Arabidopsis*, while the expression levels of the other *AtAPX* genes in the transgenic *Arabidopsis* plant were the same as or lower than those in the WT *Arabidopsis*. However, after heat stress treatment, the expression levels of some genes such as *AtAPX3*, *AtAPX6*, and *AtAPX7* were significantly up-regulated in the transgenic *Arabidopsis* plant compared with those in the WT *Arabidopsis*, indicating that the expression levels of *AtAPX3*, *AtAPX6*, and *AtAPX7* genes can be induced intensively in the transgenic *Arabidopsis* plants in response to heat stress to further enhance the scavenging ability of hydrogen peroxide in cells of plants. Taken together, these results demonstrated that the *ZmPAO6* gene can enhance heat resistance in transgenic *Arabidopsis* through modulating heat-induced H_2_O_2_ accumulation in polyamine catabolism.

## Discussion

In plants, PAs play a vital role in regulating the growth and development and protecting the plants from adverse environment including biotic and abiotic stresses. However, the catabolism of PAs in maize is not well understood. The purpose of this study is to screen key *ZmPAO* genes that are more sensitive to abiotic stresses and to lay a foundation for further revealing the regulatory mechanism of *PAO* genes in maize. Meanwhile, the possible mechanism of *ZmPAO6* gene conferring the thermotolerance in the transgenic *Arabidopsis* plants in response to heat stress was also determined in this study.

### Characterization of *PAO* Genes in Maize and Evolution of ZmPAO Proteins

An increasing number of studies have been devoted to elucidating the function of plant *PAO* genes in growth and stress responses. Until now, 5, 6, and 7 *PAO* genes have been identified in *Arabidopsis*, sweet orange, and rice, respectively (Fincato et al., 2011; Ono et al., 2012; Wang and Liu, 2015). In this study, a total of nine *ZmPAO* genes were firstly identified from the latest maize B73 genome database. The number of *PAO* genes in maize was similar to that in rice. Phylogenetic analysis demonstrated that the plant *PAO* genes were generally classified into four clades, whereas the members of maize were mainly divided into the Groups I, II, and IV but not into the Group III. Notably, the *PAO* genes in maize were closer to that in rice in phylogenetic relationship compared with those in the other species, indicating that the evolution of the *PAO* genes may occur after divergence of monocots and dicots. Furthermore, it was revealed that the result of multiple sequence alignment was consistent with that of phylogenetic relationship analysis and that among the members of the same group of PAO proteins in these plants higher identities were shared for each other, indicating that the plant PAO proteins might be highly conversed in evolution. Interestingly, AtPAO2-4 and OsPAO3-5 were localized in peroxisomes based on possessing (S/A/C)(K/R/H)(L/M) in their C-termini (Fincato et al., 2011; Ono et al., 2012). In this study, presence of SRL sequence in the C-termini of maize ZmPAO4, ZmPAO6, and ZmPAO9 and presence of CRL sequence in the C-termini of maize ZmPAO3 suggest that in the Group I, all the PAO proteins from rice, Arabidopsis and maize carry peroxisome targeting signals at their C-termini. These data suggested that these ZmPAO proteins in the Group I may be localized in peroxisomes. Notably, the *ZmPAO2* gene had no introns in gene structure, suggesting that *ZmPAO2* likely has different functions than the other *ZmPAO* genes. In addition, the analysis of the conserved domain of ZmPAO proteins was performed to confirm that all nine ZmPAO proteins contained amine-oxidase domains, indicating that these proteins were highly conserved. The analysis of gene structures and conserved motifs of ZmPAO proteins demonstrated that the members in the same subfamily have similar structures and even the same or similar motifs. The analysis of gene duplication events of *ZmPAO* genes further elaborated that two pairs of genes (*ZmPAO3*/*ZmPAO4* and *ZmPAO6*/*ZmPAO9*) could be resulted from segmental duplication during their process of evolution, indicating that segmental duplication events might play a vital role in the evolution of *PAO* gene family in maize.

### Expression Profiles Analysis of *ZmPAO* Genes in Response to Abiotic Stresses

The dynamic balance of intracellular polyamines was attributed to the regulation of PA biosynthesis and catabolism. Previous studies have shown that the dynamic balance of PAs could be challenged under adverse environmental conditions. For instance, the excessive accumulation of PAs caused by various abiotic stresses requires more PAO proteins to rebalance the dynamic balance of polyamines (Wang et al., 2011; Minocha et al., 2014). It was reported that a spaceflight induced wheat mutant, named salinity tolerance 1 (*st1*), is a salinity-tolerant line. In the salt-treated wheat mutant *st1*, genes encoding polyamine oxidase showed higher expression compared with the salt-treated WT, indicating that these genes may play a crucial role in salinity tolerance in *st1* mutant (Xiong et al., 2017). Moreover, previous studies have reported that polyamine oxidase encoding extracellular enzyme in rice may play a key role in the protection of plant cells (Sagor et al., 2021). Therefore, it is necessary to analyze the expression patterns of *ZmPAO* genes under various abiotic stresses. This study demonstrated that the expression levels of most *ZmPAO* genes were up-regulated under heat and salt treatments. In addition, except for the up-regulation of expression levels of *ZmPAO1*, *ZmPAO5*, and *ZmPAO8* genes in the early stage of stress, almost all *ZmPAO* genes in stems and leaves were down-regulated under drought treatment. These results suggested that maize *PAO* genes could be involved in various abiotic stress responses.

### Ectopic Overexpression of *ZmPAO6* Enhances Heat Tolerance in Transgenic *Arabidopsis*

To adequately comprehend the role of *PAO* genes in plant development and abiotic stresses, it is crucial to conduct in-depth research by overexpressed transgenic plants, which will be helpful in further exploring the biochemical and physiological roles of these *PAO* genes in maize. PAOs have been proved to be the pivotal enzymes in regulating the levels of intracellular PAs, and the dynamic balance of PAs is essential for plant development (Handa and Mattoo, 2010; Tiburcio et al., 2014). The stress resistance of plants mainly depends on stress-responsive genes, and the overexpression of these genes could improve the adaptability of plants to various environmental stresses (Alcázar et al., 2006). According to the phylogenetic analysis and expression patterns of *ZmPAO* genes under various abiotic stresses in this study, the *ZmPAO6* gene was selected for performing the preliminary functional verification experiments under heat stress treatment to further reveal if this newly-identified gene could function in improving heat tolerance in transgenic *Arabidopsis* plants. Thus, the *ZmPAO6* gene was overexpressed in *Arabidopsis* by genetic transformation. Under control condition, overexpression of *ZmPAO6* resulted in an increased production of H_2_O_2_. Similarly, a previous study showed that overexpression of *AtPAO3* resulted to an increased production of H_2_O_2_ in *Arabidopsis* (Andronis et al., 2014). Moreover, ectopic overexpression of *AtPAO5* enhanced the conversion of lateral root primordia into shoots and influenced the PA homeostasis during this period, and resulted in elevated ROS level in the lateral root primordia of *Arabidopsis* (Kaszler et al., 2021). Therefore, overexpression of *ZmPAO6* in transgenic *Arabidopsis* may also share similar functional effects in this study. Moreover, high temperature is a vital environmental factor that affects plant growth and crop yield (Iba, 2002). In this study, compared with the WT *Arabidopsis*, the leaves of transgenic *Arabidopsis* plants maintained a better green and stretched state. After heat stress treatment, the phenotype and physiological indicators of the WT and transgenic *Arabidopsis* plants exhibited apparent different changes. Further studies demonstrated that the transgenic *Arabidopsis* plants had better growth vitality under heat stress treatment, indicating that the overexpression of *ZmPAO6* gene in the transgenic *Arabidopsis* conferred its tolerance to heat stress. The enhancement of adaptability of these transgenic plants in response to heat stress was required for the synchronous adaptation of external morphological and biochemical levels.

In previous studies, the plant protection depended on increased levels of H_2_O_2_ produced by Spm oxidation, implying that the catabolism of PAs mainly including Spm catalyzed by PAOs has been considered to be a crucial component of the plant defense response (Gonzalez et al., 2011). In this study, under normal and heat stress conditions, *Arabidopsis* plants overexpressing the *ZmPAO6* gene showed lower Spm contents compared with WT *Arabidopsis*, indicating that *ZmPAO6* may be involved in the back-conversion reactions of PAs, which is mainly involved in the oxidation of Spm to Spd. Accordingly, the Spd content of transgenic *Arabidopsis* was higher than those of WT *Arabidopsis*. Therefore, the results in this study suggested that plant-specific PA degradation (mainly Spm) may play a vital role in plant defense. Furthermore, MDA is used as a widely marker of lipid oxidation damage at the physiological level of plants, which reflects the level of membrane lipid peroxidation in plants to measure the degree of oxidative stress (Davey et al., 2005). In this study, less accumulation of MDA was detected in the *ZmPAO6*-overexpressed transgenic *Arabidopsis* lines compared with that in the WT *Arabidopsis*, which indicated that the overexpression of *ZmPAO6* gene could maintain the stability of lipid membrane structure under heat stress treatment.

In addition, the plants are likely to regulate stress resistance by complex signal regulatory pathways under various abiotic stresses. Among them, one of the consequences of abiotic stresses is to increase the concentration of intracellular ROS (Apel and Hirt, 2004). H_2_O_2_ was initially considered to be a toxic reactive oxygen species because of its damage to a variety of cell structures. In addition, H_2_O_2_ complements, cooperates or antagonizes many cellular regulatory circuits through positive interactions with other signals and plant hormones in growth, development and stress responses (Quan et al., 2008; Petrov and Van Breusegem, 2012). H_2_O_2_ is a vital molecule involved in signal transduction and plant defense responses, which is constantly generated from a variety of sources, including PA catabolism (Moschou et al., 2008a; Cheng et al., 2015; Jasso-Robles et al., 2020). Therefore, before the experiment, we hypothesized that the H_2_O_2_ content of transgenic *Arabidopsis* was higher than that of WT *Arabidopsis* under heat stress, and this could in turn affect the heat resistance of transgenic *Arabidopsis*. However, in this study, contrary to our expectations, H_2_O_2_ contents in transgenic *Arabidopsis* lines were significantly lower than that in WT *Arabidopsis* under heat stress treatment. A previous study showed that ABA enhanced the accumulation of PA in grape and induced the PA oxidation pathway (Toumi et al., 2010). The accumulated PAs was mainly oxidized in the apoplast to produce H_2_O_2_ which integrated the signal pathway. The PAO generated apoplastic H_2_O_2_ resulting from osmotic stress acted as a secondary messenger in the signaling pathway and/or induces PCD in grape (Toumi et al., 2010). Under normal and stress conditions, maintaining the appropriate balance of PA catabolic pathways with the H_2_O_2_ dual action is helpful to clarify the adaptation mechanisms of plants (Yoda et al., 2009; Andronis et al., 2014). Therefore, we have reason to speculate that the overexpression of *ZmPAO6* gene may maintain the balance between PA catabolism and H_2_O_2_ in transgenic *Arabidopsis* under heat stress treatment. Taking together, although the H_2_O_2_ levels of WT and transgenic *Arabidopsis* plants increased significantly under heat stress treatment, the H_2_O_2_ contents in the transgenic plants were lower than those of the WT *Arabidopsis*, indicating that overexpression of *ZmPAO6* gene can result in the reduced accumulation of heat-induced H_2_O_2_ content, which suggested that maize PAO proteins might be crucial for modulating the dynamic balance of H_2_O_2_ in transgenic *Arabidopsis*.

To further explore the underlying molecular mechanism of H_2_O_2_ pathways involved in heat stress, the expression levels of glutathione peroxidase (*GPX*) and ascorbate peroxidase (*APX*) genes were investigated in this study, which belong to the main antioxidant enzymes for H_2_O_2_ scavenging and can catalyze the reduction of H_2_O_2_ to prevent the potential cell damage caused by H_2_O_2_ (Ozyigit et al., 2016). In this study, the expression levels of antioxidant enzyme genes (*AtGPX1-8* and *AtAPX1-8*) in the WT and transgenic *Arabidopsis* plants were determined under the control and heat stress treatment conditions, respectively. In this study, after heat stress treatment, the expression levels of most *AtGPX* and *AtAPX* genes in transgenic *Arabidopsis* plants were apparently greater than that of the WT *Arabidopsis*, especially the *AtGPX* genes. In addition, it has been revealed that the exogenous H_2_O_2_ increased the expression levels of *APX* genes in tomato seedlings under salinity-alkalinity stress (Xu et al., 2021). Therefore, the increased expression of most *AtAPX* and *AtGPX* genes in transgenic *Arabidopsis* lines may be resulted from the enhanced H_2_O_2_ contents exhibited in these lines. We speculate that the overexpressed *ZmPAO6* gene in transgenic *Arabidopsis* produced the corresponding polyamine oxidase to catalyze the decomposition of PA accumulated under heat stress treatment to produce H_2_O_2_. Moreover, compared with the WT *Arabidopsis*, the expression levels of antioxidant enzymes genes in transgenic plants were up-regulated. Studies have shown that all eight *APX* genes of *Arabidopsis* participated in the processes of growth and development and abiotic stress response (Li et al., 2019). Recent evidence suggested that GPXs can protect cells from stress-induced oxidative damages and function as a vital component of growth and development in plants (Bela et al., 2015). Moreover, the accumulation of ROS is regulated by a complex antioxidant scavenging system, including GRX, SOD, APX, etc., that function as antioxidants and allow the transmission of oxidative signals (Singh et al., 2022). Therefore, these data suggested that increased the activity of some key antioxidant enzymes and reduced heat-induced H_2_O_2_ accumulation, thus enhancing stress resistance of the transgenic plants. In this study, the H_2_O_2_ contents of transgenic *Arabidopsis* plants were lower than those of WT *Arabidopsis*, perhaps due to the increased activity of AtAPX and AtGPX enzymes, which is likely to scavenge excess H_2_O_2_. However, further studies are required for clarifying whether the increased expression levels of these antioxidant enzyme genes control the H_2_O_2_ content or whether the H_2_O_2_ content controls the induction of these genes or enzymes. Although it may be involved in some complicated metabolic processes, it is worth studying carefully to further understand the regulation of H_2_O_2_ homeostasis. In conclusion, alterations in PA catabolism affect the levels of H_2_O_2_ and the expression levels of *AtAPX* and *AtGPX* genes that enhance the antioxidant defense of the plants, suggesting a link between the *ZmPAO6* gene and other enzymes involved in H_2_O_2_ homeostasis in transgenic *Arabidopsis*, and indicating that this gene can enhance heat resistance in transgenic *Arabidopsis* through modulating heat-induced H_2_O_2_ accumulation in polyamine catabolism.

**Figure 12:**
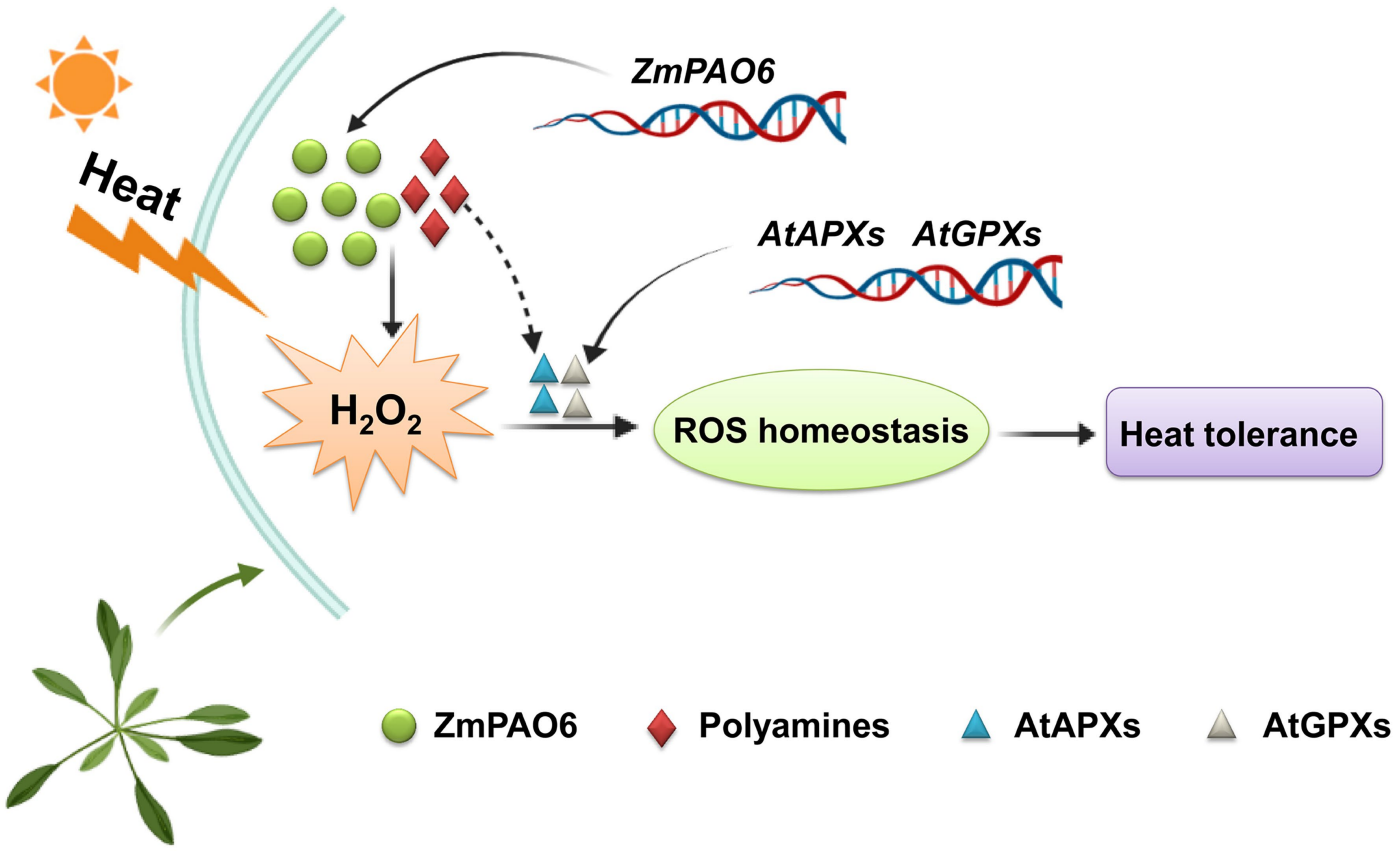
A model for the role of the *ZmPAO6* gene under heat stress in transgenic *Arabidopsis*.

Furthermore, we proposed a model of the *ZmPAO6* gene involved in PA catabolism and H_2_O_2_ homeostasis in transgenic *Arabidopsis* (Figure 12). Under heat stress treatment, the expression level of *ZmPAO6* gene was up-regulated, and the corresponding activity of polyamine oxidase was also increased, which may promote the decomposition of PAs (mainly Spm) to produce H_2_O_2_. In addition, H_2_O_2_ may be used as a signal molecule to increase the expression levels of *AtAPX* and *AtGPX* genes to accelerate the degradation of H_2_O_2_. On the other hand, heat stress causes the accumulation of H_2_O_2_ in transgenic *Arabidopsis*, which may also act on related antioxidant enzyme genes. In addition, *Arabidopsis* plants overexpressing the *ZmPAO6* gene showed increased the expression levels of *AtAPX* and *AtGPX* genes, which represented an attempt to scavenge excess H_2_O_2_ generated from the oxidation of PAs catalyzed by potential ZmPAO6 protein. However, the exact mechanism is unknown for maintaining ROS homeostasis in regulating the heat stress response in transgenic *Arabidopsis*. Therefore, the positive regulatory mechanism of *ZmPAO6* gene in response to heat stress remains to be further elucidated in future.

## Conclusion

In our study, a total of nine *PAO* genes were identified in maize, which were distributed on five chromosomes at different densities. These PAO proteins were mainly categorized into three distinct subfamilies based on the similarities of the amino acid sequences. The motif composition and exon/intron distribution were considerably conserved among the members of the same group or subgroup. The close phylogenetic relationship among PAO proteins of different species in the same subgroup provides insights into their putative functions. A comprehensive expression profile of all members of *PAO* gene family in maize suggests that these genes play functional developmental roles in different tissues. Furthermore, the expression profiles of *ZmPAO* genes were up-regulated or down-regulated under three various abiotic stress treatments, indicating that these *PAO* genes may play a crucial role in resisting adverse environment of maize. Eventually, the functional analysis of the maize representative *ZmPAO6* gene in response to heat stress demonstrated that overexpression of *ZmPAO6* in transgenic *Arabidopsis* can confer enhanced heat tolerance through modulating heat-induced H_2_O_2_ accumulation in polyamine catabolism. Taken together, our results are the first to report the *ZmPAO6* gene response to heat stress in plants and will serve to present an important theoretical basis for further unraveling the function and regulatory mechanism of *ZmPAO* genes in growth, development and adaptation to abiotic stresses in maize.

## Data Availability Statement

The original contributions presented in the study are included in the article/Supplementary Material, further inquiries can be directed to the corresponding author.

## Ethics Statement

These methods were carried out in accordance with relevant guidelines and regulations including the IUCN Policy Statement on Research Involving Species at Risk of Extinction and the Convention on the Trade in Endangered Species of Wild Fauna and Flora. We confirm that all experimental protocols were approved by Anhui Agriculture University and Anhui Normal University.

## Author Contributions

YQ, YX, WH, YZ, and XL designed the studies, carried out all the experimental analyses, and prepared all figures and tables. YX and WH drafted the manuscript. YQ assisted in explaining the results and revised the final manuscript. All authors contributed to the article and approved the submitted version.

## Funding

This study was supported by grants from the National Natural Science Foundation of China (NSFC; grant no. 31571673) and the open fundings of National Engineering Laboratory of Crop Stress Resistance Breeding (grant no. KNZJ1023) and Anhui Provincial Key Laboratory of the Conservation and Exploitation of Biological Resources (grant no. Swzy202003) and Anhui Provincial Academic Funding Project for Top Talents in Disciplines (Majors; grant no. gxbjZD2021044). The funders had no role in the study design, collection, analysis, and interpretation of data, or in the writing of the report or decision to submit the article for publication.

## Conflict of Interest

The authors declare that the research was conducted in the absence of any commercial or financial relationships that could be construed as a potential conflict of interest.

## Publisher’s Note

All claims expressed in this article are solely those of the authors and do not necessarily represent those of their affiliated organizations, or those of the publisher, the editors and the reviewers. Any product that may be evaluated in this article, or claim that may be made by its manufacturer, is not guaranteed or endorsed by the publisher.
