# Supplementary material for: Genome-Wide Identification and Functional Analysis of Polyamine Oxidase Genes in Maize Reveal Essential Roles in Abiotic Stress Tolerance
Source: Front Plant Sci. 2022 Aug 4;13:950064. doi: 10.3389/fpls.2022.950064 (PMC9386529; doi:10.3389/fpls.2022.950064)
Supplement: Supplementary file 1 [file Data_Sheet_1.DOCX]

Supplementary Material

## Supplementary Figures


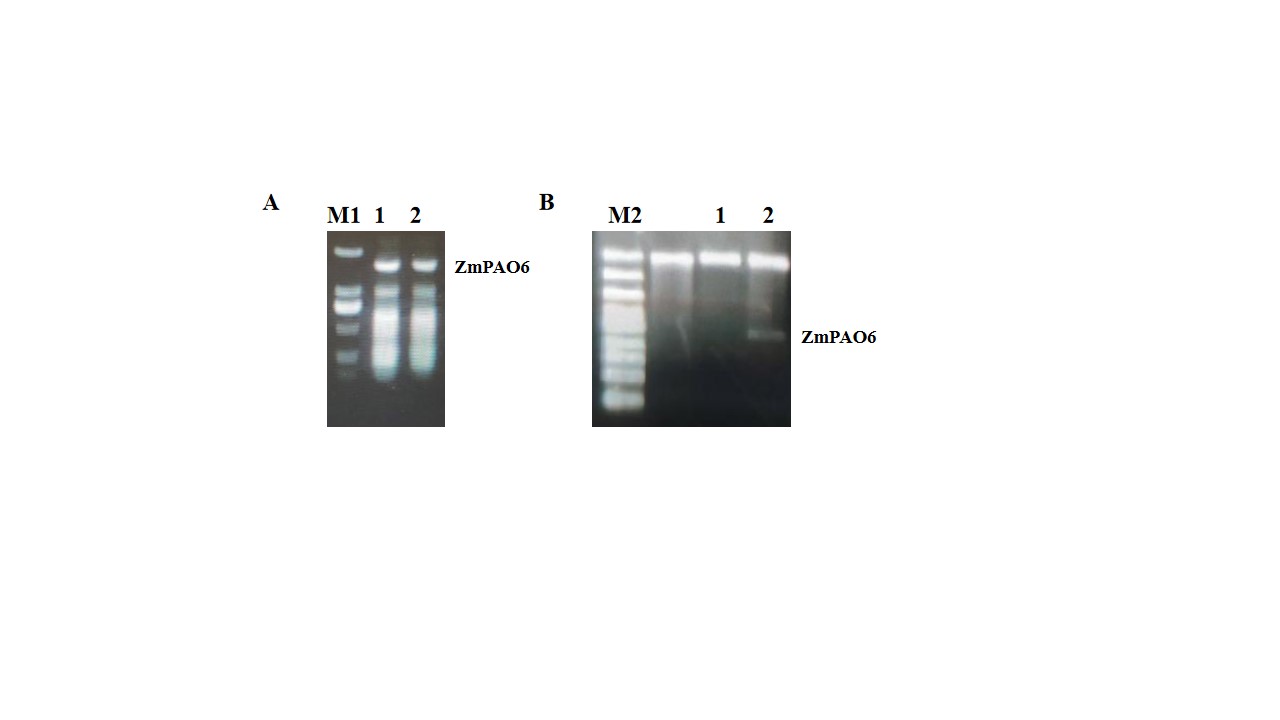


**Supplementary Figure 1. (A)** Amplification of *ZmPAO6* via RT-PCR. M1, 250-Ⅰ Marker; A1-2, *ZmPAO6*. **(B)** Recombinant plasmid. M2, 250-Ⅲ Marker; B1, Empty vector; B2, Recombinant plasmid.


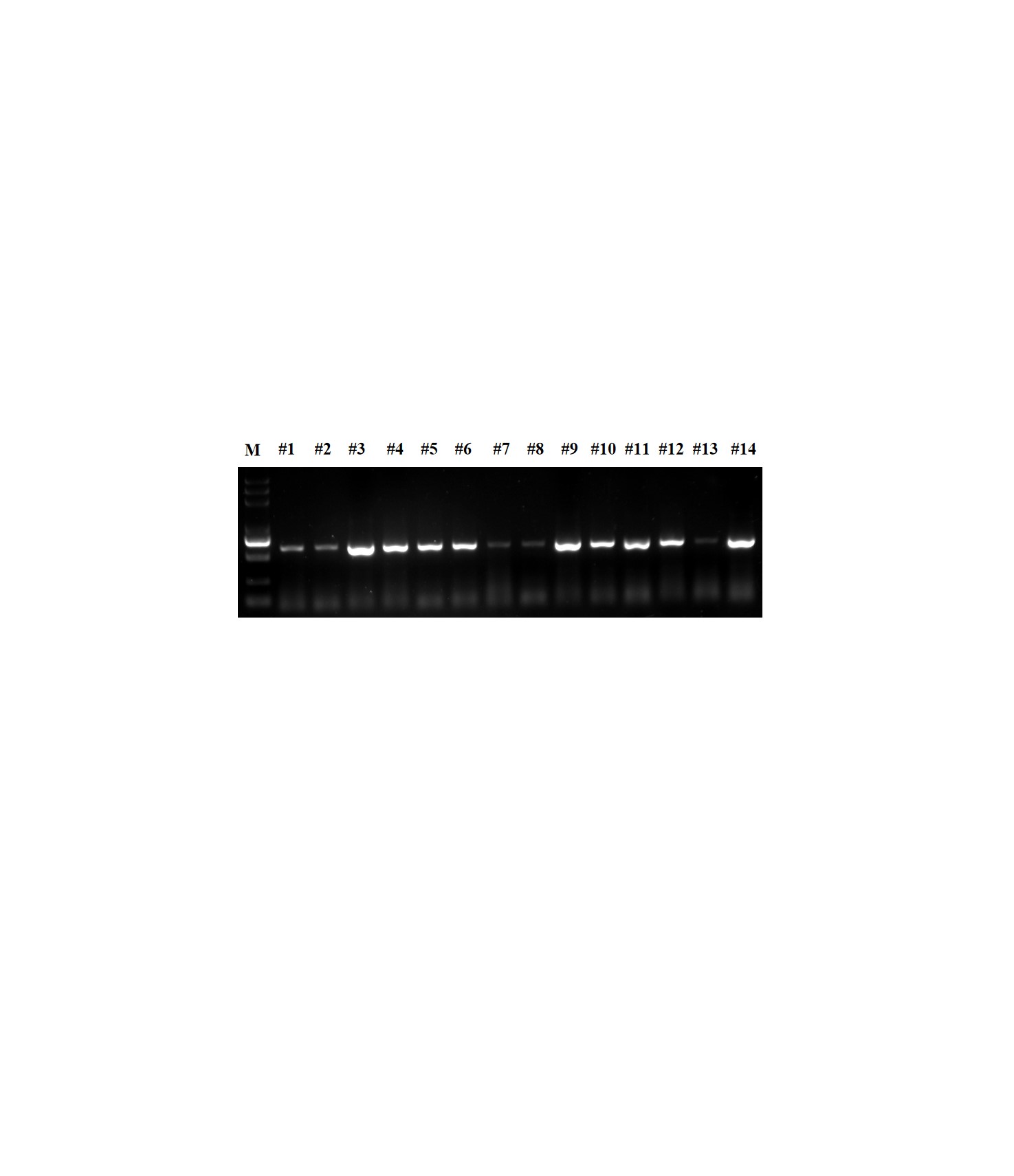


**Supplementary Figure 2.** Detection of the hygromycin resistance gene by PCR. Marker：5k, 3k, 2k, 1000, 750, 500, 250, 100bp.


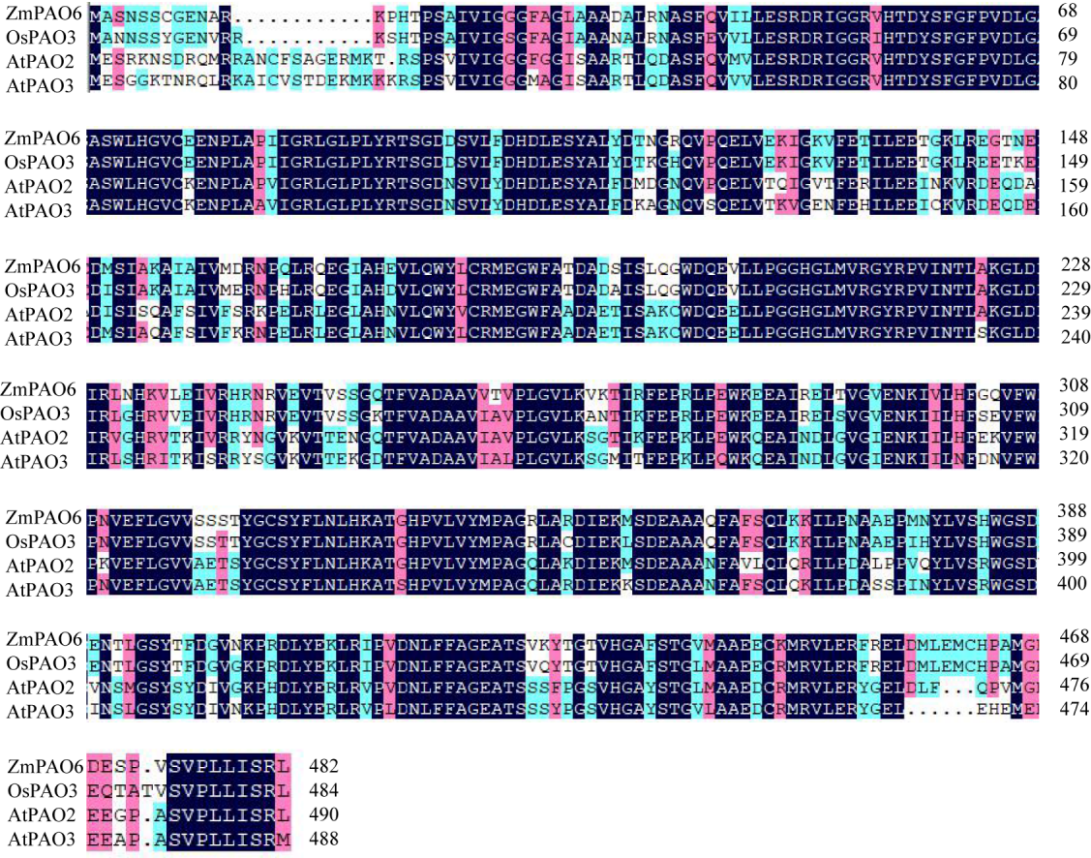


**Supplementary Figure 3.** Alignment of the amino acid sequences of PAO proteins in plants.

**2 Supplementary Tables**

**Supplementary Table 1 The information of PAO genes in plants**

| **Name** | Gene ID |
| --- | --- |
| *ZmPAO7* | Zm00001d037642_T001 |
| *ZmPAO9* | Zm00001d002266_T001 |
| *ZmPAO6* | Zm00001d026334_T001 |
| *ZmPAO8* | Zm00001d036513_T001 |
| *ZmPAO4* | Zm00001d026586_T003 |
| *ZmPAO3* | Zm00001d001883_T001 |
| *ZmPAO2* | Zm00001d043681_T001 |
| *ZmPAO1* | Zm00001d024281_T001 |
| *ZmPAO5* | Zm00001d028172_T001 |
| *AtPAO1* | AT5G13700.1 |
| *AtPAO2* | AT2G43020.1 |
| *AtPAO3* | AT3G59050.1 |
| *AtPAO4* | AT1G65840.1 |
| *AtPAO5* | AT4G29720.1 |
| *OsPAO1* | LOC_Os01g51320 |
| *OsPAO2* | LOC_Os03g09810 |
| *OsPAO3* | LOC_Os04g53190 |
| *OsPAO4* | LOC_Os04g57550 |
| *OsPAO5* | LOC_Os04g57560 |
| *OsPAO6* | LOC_Os09g20260 |
| *OsPAO7* | LOC_Os09g20284 |
| *VvPAO1* | VIT_01s0127g00750 |
| *VvPAO2* | VIT_01s0127g00800 |
| *VvPAO3* | VIT_03s0017g01000 |
| *VvPAO4* | VIT_04s0043g00220 |
| *VvPAO5* | VIT_12s0028g01120 |
| *VvPAO6* | VIT_12s0055g00480 |
| *VvPAO7* | VIT_13s0019g04820 |
| *HvPAO1* | AJ298131.1 |
| *HvPAO2* | AJ298132.1 |
| *SlPAO1* | Solyc01g087590 |
| *SlPAO2* | Solyc07g043590 |
| *SlPAO3* | Solyc12g006370 |
| *SlPAO4* | Solyc02g081390 |
| *SlPAO5* | Solyc03g031880 |
| *SlPAO6* | Solyc03g031880 |
| *SlPAO7* | Solyc05g018880 |
| *CsPAO1* | Cs7g02060.1 |
| *CsPAO2* | Cs7g18840.2 |
| *CsPAO3* | Cs6g15870.1 |
| *CsPAO4* | Cs4g14150.1 |
| *CsPAO5* | Cs7g23790.1 |
| *CsPAO6* | Cs7g23670.1 |
| *GhPAO1* | A05G029900.1 |
| *GhPAO2* | A07G011100.1 |
| *GhPAO3* | A08G037950.1 |
| *GhPAO4* | A08G055400.1 |
| *GhPAO5* | A08G143000.1 |
| *GhPAO6* | D12G092000.1 |
| *GhPAO7* | A05G031500.1 |
| *GhPAO8* | A07G012700.1 |
| *GhPAO9* | A08G037900.1 |
| *GhPAO10* | A08G143000.1 |
| *GhPAO11* | A08G055400.1 |
| *GhPAO12* | A12G092200.1 |

**Supplementary Table 2 The signal values for maize *PAO* genes in the ten tissues**

| **Name** | **Gene ID** | **Seed** | **Root** | **Seedling** | **Stem** | **Shoot Tip** | **Silks** | **Leaf** | **Tassel** | **Husk** | **Endosperm** |
| --- | --- | --- | --- | --- | --- | --- | --- | --- | --- | --- | --- |
| ZmPAO1 | GRMZM2G034152 | 6.45 | 12.45 | 15.19 | 12.99 | 13.42 | 6.05 | 14.13 | 12.04 | 14.29 | 5.12 |
| ZmPAO2 | GRMZM2G035994 | 6.35 | 6.41 | 6.73 | 6.51 | 6.63 | 6.96 | 6.97 | 6.55 | 6.44 | 6.44 |
| ZmPAO3 | GRMZM2G078033 | 11.48 | 8.22 | 10.57 | 9.99 | 9.79 | 10.12 | 11.12 | 10.27 | 11.68 | 12.17 |
| ZmPAO4 | GRMZM2G150248 | 9.22 | 12.79 | 11.19 | 10.26 | 9.63 | 12.5 | 11.56 | 11.57 | 11.24 | 10.79 |
| ZmPAO5 | GRMZM2G071343 | 5.22 | 4.83 | 5.4 | 4.76 | 4.93 | 5.5 | 4.58 | 4.94 | 4.77 | 5.8 |
| ZmPAO6 | GRMZM2G396856 | 9.71 | 11.16 | 9.78 | 11.32 | 10.07 | 10.47 | 10.2 | 10.29 | 10.47 | 10.1 |
| ZmPAO7 | AC216196.3 | 8.59 | 6.46 | 5.4 | 5.98 | 5.03 | 4.82 | 5.1 | 5.29 | 4.93 | 7.5 |
| ZmPAO8 | AC206319.2 | 5.66 | 6.24 | 6.11 | 5.89 | 5.88 | 6.25 | 6.29 | 6.23 | 5.85 | 6.04 |
| ZmPAO9 | GRMZM2G000052 | 11.29 | 13.7 | 12.86 | 12.71 | 12.54 | 13.42 | 12.57 | 11.42 | 13.04 | 11.27 |

**Supplementary Table 3 Primer sequences for RT-qPCR in this study**

| **Name** | **Gene ID** | **F-primer** | **R-primer** |
| --- | --- | --- | --- |
| ZmPAO1 | Zm00001d024281 | GTGGACTACTACAAGTTCGACT | GTCGACGATCTTTCCAGACTTA |
| ZmPAO2 | Zm00001d043681 | CTTCCTGGGATCCTACACCTA | AAGTCTGCAACCTAAGCGTAG |
| ZmPAO3 | Zm00001d001883 | GACCTGAGTGAGATTGGATCAC | AAAAAGACTCGGCAACCATCAG |
| ZmPAO4 | Zm00001d026586 | CCGGTTTGTTGCTTGATGTAAT | GACGTTTACCTGACGAAAAACA |
| ZmPAO5 | Zm00001d028172 | CTGTCTTAGCCATGCAACGG | AAGTAGTTGTCCTCGCCGAA |
| ZmPAO6 | Zm00001d026334 | CCTTCTCCACTGGTGTTATGG | TAGACAACCTCTCGTCTGCTTA |
| ZmPAO7 | Zm00001d037642 | ACAGTTGAGGCAAGAAGGGA | TTCCGGTGGCGAACAATTTC |
| ZmPAO8 | Zm00001d036513 | TTCTGTTGGGTGTCCTGAAAGCG | TCTCAATGTCACGAGCAAGTCGG |
| ZmPAO9 | Zm00001d002266 | TGTGACATGTAACACCCTTACA | CGGCGAGCCATTTAACTAAT |
| Zm-actin | GQ339773.1 | CACAATGTTTGGCGGGATTGGTGA | TGTACTTCCTTTCCGGTGGAGCAA |
| APX1 | AT1G07890 | GATGTCTTTGCTAAGCAGATGG | GAGTTGTCGAAGATTAGAGGGT |
| APX2 | AT1G77490 | GAAATAGTTGCCTTGTCTGGTG | CATTTCACTGTCCATGACTGTC |
| APX3 | AT3G09640 | AAGGCTCTTCTTGATGATCCTC | GCTTCCGTATAGTCTTCGAAGA |
| APX4 | AT4G08390 | TGAGACGAAGTACACGAAAGAA | CCTGATCTGCAGCATACTTTTC |
| APX5 | AT4G09010 | TTGCTTTCAACCACCATTAAGG | TGCATATGAAATAGGACCTCCC |
| APX6 | AT4G32320 | GACTGCTTCTCTGGTTAAGACT | CCTTCTACTTGCAACGAAAACA |
| APX7 | AT4G35000 | GCAAAGGATGAAGATGCATTCT | TCCGGATCTCGTAAAAGTAACC |
| APX8 | AT4G35970 | GAACTCTCTTCTCTCGTATGGG | TAGAGCCTTGTCGGTTTTAAGT |
| GPX1 | AT1G63460 | TTCCAAATGTGGGATGACAAAC | GGGAATTCAGATTTGAAGCGAG |
| GPX2 | AT2G25080 | TCTCTTCAAGTCTCGTCCTTTC | ATGTCAAACCACATCTTGAAGC |
| GPX3 | AT2G31570 | GGACCAATACAAAGGCAAAACT | GTGCAGACAGTTTGTTGAATCT |
| GPX4 | AT2G43350 | AATGTAGCCTCTAAGTGTGGTC | CCCAAACAATCCTCCTTTTTGT |
| GPX5 | AT2G48150 | CAAAACGCAGCACCAATCTATA | TTCTCGATTGATAGCGGTGTAA |
| GPX6 | AT3G63080 | AAGAGGCTCATCAATTTGCTTG | TTGTGCCATAACGATCAATGAC |
| GPX7 | AT4G11600 | GGAATCAAGAGCCTGGTACTAA | TTTGTCACCGTTAACATCAACC |
| GPX8 | AT4G31870 | GCAGAGTTCCCTATATTCGACA | CTCGACAACTTTGCCCTTTTTA |
| At-actin | AT3G18780 | CTTGCACCAAGCAGCATGAA | CCGATCCAGACACTGTACTTCCTT |
